# New algorithms for structure informed genome rearrangement

**DOI:** 10.1186/s13015-023-00239-x

**Published:** 2023-12-01

**Authors:** Eden Ozeri, Meirav Zehavi, Michal Ziv-Ukelson

**Affiliations:** Department of Computer Science, Ben Gurion University of the Negev, Be’er Sheva, Israel

**Keywords:** PQ-tree, Gene cluster, Breakpoint distance

## Abstract

We define two new computational problems in the domain of perfect genome rearrangements, and propose three algorithms to solve them. The rearrangement scenarios modeled by the problems consider Reversal and Block Interchange operations, and a PQ-tree is utilized to guide the allowed operations and to compute their weights. In the first problem, $\mathsf {Constrained \ TreeToString \ Divergence}$ ($\textsf{CTTSD}{}$), we define the basic structure-informed rearrangement measure. Here, we assume that the gene order members of the gene cluster from which the PQ-tree is constructed are permutations. The PQ-tree representing the gene cluster is ordered such that the series of gene IDs spelled by its leaves is equivalent to that of the reference gene order. Then, a structure-informed genome rearrangement distance is computed between the ordered PQ-tree and the target gene order. The second problem, $\mathsf {TreeToString \ Divergence}$ ($\textsf{TTSD}{}$), generalizes $\textsf{CTTSD}{}$, where the gene order members are not necessarily permutations and the structure informed rearrangement measure is extended to also consider up to $d_S$ and $d_T$ gene insertion and deletion operations, respectively, when modelling the PQ-tree informed divergence process from the reference gene order to the target gene order. The first algorithm solves $\textsf{CTTSD}{}$ in $O(n \gamma ^2 \cdot (m_p \cdot 1.381^\gamma + m_q))$ time and $O(n^2)$ space, where $\gamma$ is the maximum number of children of a node, *n* is the length of the string and the number of leaves in the tree, and $m_p$ and $m_q$ are the number of P-nodes and Q-nodes in the tree, respectively. If one of the penalties of $\textsf{CTTSD}$ is 0, then the algorithm runs in $O(n m \gamma ^2)$ time and $O(n^2)$ space. The second algorithm solves $\textsf{TTSD}{}$ in $O(n^2 \gamma ^2 {d_T}^2 {d_S}^2\,m^2 (m_p \cdot 5^\gamma \gamma + m_q))$ time and $O(d_T d_S m (m n + 5^\gamma ))$ space, where $\gamma$ is the maximum number of children of a node, *n* is the length of the string, *m* is the number of leaves in the tree, $m_p$ and $m_q$ are the number of P-nodes and Q-nodes in the tree, respectively, and allowing up to $d_T$ deletions from the tree and up to $d_S$ deletions from the string. The third algorithm is intended to reduce the space complexity of the second algorithm. It solves a variant of the problem (where one of the penalties of $\textsf{TTSD}$ is 0) in $O(n \gamma ^2 {d_T}^2 {d_S}^2\,m^2 (m_p \cdot 4^{\gamma }\gamma ^2n(d_T+d_S+m+n) + m_q))$ time and $O(\gamma ^2 n m^2 d_T d_S (d_T+d_S+m+n))$ space. The algorithm is implemented as a software tool, denoted MEM-Rearrange, and applied to the comparative and evolutionary analysis of 59 chromosomal gene clusters extracted from a dataset of 1487 prokaryotic genomes.

## Introduction

Recent advances in pyrosequencing techniques, combined with global efforts to study infectious diseases, yield huge and rapidly-growing databases of microbial genomes [1, 2]. These big new data statistically empower genomic-context based approaches to functional and evolutionary analysis: the biological principle underlying such analyses is that groups of genes that are located close to each other across many genomes often code for proteins that interact with one another, suggesting a common functional association.

Groups of genes that are co-locally conserved across many genomes are denoted *gene clusters*. The order of the genes in distinct genomic occurrences of a gene cluster may not be conserved. A specific order of the genes of a gene cluster, that is co-linearly conserved across many genomes, is denoted a *gene order* of the gene cluster. The distinct genomes in which a gene order occurs are denoted *instances* of the gene order. Gene clusters in prokaryotic genomes often correspond to (one or several) operons; those are neighbouring genes that constitute a single unit of transcription and translation.

In this paper, our biological goal is to study the evolution of gene clusters in prokaryotes, by computing the divergence between pairs of gene orders that belong to the same gene cluster, based on genome rearrangement scenarios. When defining this computational task as an optimization problem, one needs to take into account that parsimony considerations may not be sufficient: driven by the objective to keep the genome small and efficient, in spite of the high rate of gene shuffling in the prokaryotic genome, only gene orders that are reinforced by conveying some advantage in fitness (i.e., in adaptation to some niche) will be kept in the genome. This calls for a Structure-Informed Genome Rearrangement (SIGR) divergence measure that will interleave parsimony considerations with some learned structural and functional information regarding the gene cluster under study. Such a measure could more accurately assess the degree of divergence from one order of a gene cluster to another, and provide further understanding of gene-context level environmental-specific adaptations [3, 4].

To this end, we propose a new SIGR-based divergence measure and provide efficient algorithms to compute it. According to our approach, information regarding the structure of the gene cluster is learned from the known gene orders of the gene cluster and represented by a PQ-tree (formally defined in “Preliminaries” section). The PQ-tree is then utilized to both guide the allowed operations and to compute their weights. A motivating example for our proposed approach, including exemplifying figures, can be found in “A motivating example” section.

PQ-trees have been advocated as a representation for gene clusters [5–7]. A PQ-tree describes the possible permutations of a given sequence, and can be constructed in polynomial-time [8]. The PQ-tree representing a given gene cluster describes its hierarchical inner structure and the relations between instances of the gene cluster succinctly, assists in predicting the functional association between the genes in the gene cluster, yields insights into the evolutionary history of the gene cluster, and provides a natural and meaningful way of visualizing complex gene clusters.

The biological assumptions underlying the representation of gene clusters as PQ-trees is that operons evolve mainly via progressive merging of sub-operons, where the most basic units in this recursive operon assembly are colinearly conserved sub-operons [9]. In the case where an operon is assembled from sub-operons that are colinearly dependent, the conserved gene order could correspond, e.g., to the order in which the transcripts of these genes interact in the metabolic pathway in which they are functionally associated [10]. Thus, rearrangement events that shuffle the order of the genes (or of smaller sub-operons) within this sub-operon could affect the function of its product. On the other hand, inversion events in which the genes participating in this sub-operon remain colinearly ordered with respect to the transcription order, have less of an affect on the interactions between the sub-operon’s gene products.

The case of colinearly conserved sub-operons is represented in the PQ-tree by a Q-node (marked with a rectangle in the exemplifying figures), and by a *Reversal* operation in the corresponding pairwise gene order rearrangement scenario. In the case where an operon is assembled from sub-operons that are not colinearly co-dependent, convergent evolution could yield various orders of the assembled components [9]. This case is represented in the PQ-tree by a P-node (marked with a circle in the exemplifying figures), and by a *Block Interchange* operation in the corresponding pairwise gene order rearrangement scenario.

### Background on structure informed genome rearrangement (SIGR) scenarios

A generic formulation of genome rearrangement problems is, given two genomes and some allowed edit operations, to transform one genome into the other using a minimum number of edit operations [11–14]. A famous algorithmic result related to genome rearrangements concerns the problem of sorting signed permutations by Reversals. This problem aims at computing a shortest sequence of Reversals that transforms one signed permutation into another, and can be solved in polynomial time [15–17]. It was later generalized to handle, still in polynomial time, multichromosomal genomes with linear chromosomes, using rearrangements such as Translocations, Chromosome Fusions and Fissions [18, 19]. Then, a general operation called Double Cut-and-Join (DCJ), was introduced in [20] for handling problem instances where the common intervals are organized in a nonlinear structure. A DCJ can be, among others, a Reversal, a Translocation, a Fusion or a Fission, but two consecutive DCJ operations can also simulate a Block-Interchange or a Transposition.

Previous works proposed related forms of SIGR by considering rearrangement scenarios on two permutations that preserve their common intervals (groups of co-localized genes). Such scenarios, which may not be shortest among all scenarios, are called perfect [21]. Computing a Reversal scenario of minimum length that preserves a given subset of the common intervals of two signed permutations is NP-hard [21] and several papers have explored this problem, describing families of instances that can be solved in polynomial time [22–25], fixed parameter tractable algorithms [23, 26], and an exponential time algorithm (which works also in the more general weighted case) [27]. We note that all the perfect scenarios mentioned above considered only Reversal operations, while for our settings Block-Interchange operations should also be considered. A heuristic approach that implements, among others perfect reversals and block interchange is described in [28].

In [29] the notion of perfect scenario was extended to the Perfect DCJ model, thus capturing additional operations in perfect scenarios, including Cut, Join and Block-Interchange. When considering the perfect rearrangement scenarios that best fit our problem, this is the model that is most relevant to our settings, as the other (non-heuristic) previous works do not include the Block-Interchange operation. The operations considered by the Perfect DCJ model are very general, which renders the problem computationally intractable in its general setting. Indeed, Berard et al. [29] thus only obtain positive algorithmic results for special cases that, in particular, do not encompass the structure of a PQ-tree, and with a parameter that can often be of the magnitude of the entire input size. For us, the aforementioned special cases are too restricted, and cannot model the problem we have at hand. On the other hand, fortunately, for us, considering Cut and Join operations is an unjustified overhead. Specifically, we seek to model the considered evolutionary scenarios by a formulation that is more specific to our biological problem, in order to increase the divergence measure accuracy as well as tighten the parameters driving the complexity of the algorithms for the problem. Since, in our problem, we are dealing with prokaryotic gene clusters and the data in our experiment is typically confined to one chromosome per genome, we need not consider Cut and Join operations. In addition, the intervals in prokaryotic gene clusters follow a strongly conserved hierarchy, naturally modeled by the PQ-tree learned from the members of the gene cluster. In terms of divergence measure accuracy, we would like to enforce the PQ-tree structure as a constraint to the considered rearrangements. Furthermore, while the Perfect DCJ model is unweighted (and simply counts the number of DCJ operations applied), we use the PQ-tree as a guide affecting the weights of the applied rearrangement operations. In terms of tightening the parameters driving the complexity of the computation, the PQ-tree constraint enables us to use dynamic programming algorithms and to reduce the parameter from *n* to the out degree of the tree. In particular, this means that the more hierarchical the input is, the smaller our parameter is likely to be, and the faster our algorithm is—in other words, the running time of our algorithm naturally scales with the amount of structure given by the PQ-tree.

In general, our algorithm is intended for widely conserved prokaryotic gene clusters (with cluster length bounded by tens of genes) that display a hierarchical structure. In such cases, the maximal degree of a P-node (which is the exponential factor in the time complexity of our proposed algorithm) is typically small, in particular with respect to the maximal degree of a Q-node (which affects the time complexity of the algorithm by a polynomial factor.) This is due to the fact that gene clusters in bacteria are very highly colinearly conserved [30, 31], even when the benchmark dataset is large and spans a wide taxonomical range of prokaryotes [32]. Furthermore, the very same data set used in this study (consisting of 59 prokaryotic genomes) was previously analyzed by Svetlitsky et al. [32] in order to study the degree of conserved collinearity among widely spread gene clusters in prokaryotic genomes. The study found a negative correlation between the proportion of shuffled gene clusters and their length, i.e. the longer the gene cluster is, the more it is colinearly conserved. All this inspired us to develop an algorithm that, on one hand captures the structure of gene clusters in an informed way, while on the other hand employs the P-node out-degree as a bound on its exponential factor. To this end we propose an FPT algorithm for the TTSD problem, where the structure of the gene cluster is represented by a PQ-tree, and the parameter binding the exponential factor of the algorithm is the maximal out-degree of a P-node in the tree.

### Our contribution

We propose a new, two-step approach to SIGR: In the first step, given the gene orders of the gene cluster under study, the internal topology properties of a gene cluster are learned from its corresponding gene orders and a PQ-tree is constructed accordingly. Then, in the second step, given a reference gene order and a target gene order, a SIGR scenario is computed from the reference to the target, such that colinear dependencies among genes and between sub-operons, as learned by the PQ-tree, are taken into account by the penalties assigned to the rearrangement operations.

To this end, we define two new theoretical problems and propose three algorithms to solve them. In the first problem, denoted $\mathsf {Constrained \ TreeToString \ Divergence}$ ($\textsf{CTTSD}{}$), we define the basic SIGR divergence measure. Here, we assume that the gene order members of the gene cluster from which the PQ-tree is constructed are permutations. The rearrangement operations considered by this problem include (weighted) Reversals and Block-Interchange operations. In this problem, the PQ-tree representing the gene cluster (Fig. 3A) is ordered such that the series of gene IDs spelled by its leaves is equivalent to the reference gene order (Fig. 3B). Then, a weighted SIGR measure is computed from the ordered PQ-tree to the target gene order (Fig. 3C).

The second problem, denoted $\mathsf {TreeToString \ Divergence}$ ($\textsf{TTSD}{}$), is a generalization of the first problem, where the gene order members are not necessarily permutations and the genome rearrangement measure is extended to also consider up to $d_S$ gene insertion operations and up to $d_T$ gene deletion operations.

The first fixed parameter tractable (FPT) algorithm (in “Constrained TreeToString Divergence: algorithm” section) solves $\textsf{CTTSD}{}$ in $O(n \gamma ^2 \cdot (m_p \cdot 1.381^\gamma + m_q))$ time and $O(n^2)$ space, where $\gamma$ is the maximum number of children of a node, *n* is the length of the string and the number of leaves in the tree, and $m_p$ and $m_q$ are the number of P-nodes and Q-nodes in the tree, respectively. If one of the penalties of $\textsf{CTTSD}$ is defined to be 0, then the algorithm runs in $O(n m \gamma ^2)$ time and $O(n^2)$ space.

The second FPT algorithm solves $\textsf{TTSD}{}$ in $O(n^2 \gamma ^2 {d_T}^2 {d_S}^2\,m^2 (m_p \cdot 5^\gamma \gamma + m_q))$ time and $O(d_T d_S m (m n + 5^\gamma ))$ space, where $\gamma$ is the maximum number of children of a node, *n* is the length of the string, *m* is the number of leaves in the tree, $m_p$ and $m_q$ are the number of P-nodes and Q-nodes in the tree, respectively, and allowing $d_T$ deletions from the tree and $d_S$ deletions from the string.

Dynamic programming is common on trees, and on sequences, and our algorithms combine the two types. While our first algorithm is simple and intuitive (based on one dynamic programming and two greedy procedures), for our second algorithm (based on three dynamic programming procedures), more technical ingredients are required. For example, one challenge is the need to compute a vertex cover in a graph that is not fully known by any single entry of our dynamic programming table. Specifically, when we consider a single entry, some of the relevant vertices are not yet processed, and for those that are processed, we cannot store enough information (for the sake of efficiency) so as to deduce which edges exist between them.

The third FPT algorithm is intended to reduce the space complexity of the second algorithm. It solves a variant of the problem (where one of the penalties of $\textsf{TTSD}$ is defined to be 0) in $O(n \gamma ^2 {d_T}^2 {d_S}^2\,m^2 (m_p \cdot 4^{\gamma }\gamma ^2n(d_T+d_S+m+n) + m_q))$ time and $O(\gamma ^2 n m^2 d_T d_S (d_T+d_S+m+n))$ space. This algorithm employs the principle of inclusion–exclusion for the sake of space reduction, which, to the best of our knowledge, is not commonly used in the study of problems in computational biology.

The proposed general algorithm is implemented as a software tool, denoted MEM-Rearrange, and applied to the comparative and evolutionary analysis of 59 chromosomal gene clusters extracted from a dataset of 1487 prokaryotic genomes (in “Results” section). Our preliminary results, based on the analysis of the 59 gene clusters, indicate that our proposed measure correlates well with an index that is computed by comparing the class composition of the genomic instances of the two compared gene orders. The correlations yielded by our measure are shown to significantly increase with the increase in conserved structure of the corresponding gene clusters (as modelled by PQ-trees).

### Roadmap

The rest of the paper is organized as follows. In “A motivating example” section we present a motivating biological example. Previous works are reviewed in “Previous related works” section. In “Preliminaries” section, we formally define the terminology used throughout the paper, and, in particular, the two problems studied in this paper. In “Constrained TreeToString Divergence: algorithm” section, we present our first algorithm, which solves the $\textsf{CTTSD}{}$ problem. In “TreeToString Divergence: algorithm” section, we present our second algorithm, which solves the $\textsf{TTSD}{}$ problem. In “TTSD: polynomial space complexity” section, we present our third algorithm, which solves the $\textsf{CTTSD}{}$ problem and improves the space complexity of the second algorithm. In “Methods and datasets” section, we specify the details of our data set construction and experiment. Finally, in “Results” section, we compare the performance of our proposed rearrangement measure versus that of signed break-point distance on a benchmark of 59 chromosomal gene clusters. Concluding remarks are given in “Conclusions” section.

## A motivating example

**Fig. 1:**
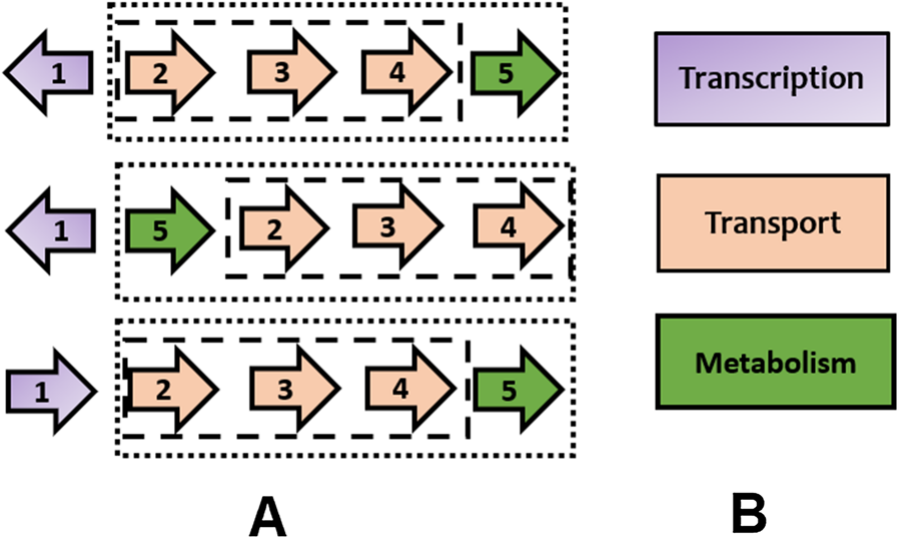
Gene cluster of a known sugar transporter operon. **A** Three gene order instances of the gene cluster. **B** The color-coding key for the functional categories of the genes

In this section we give a biological example to motivate the new problems defined in this paper. To this end, we chose to interpret the first result shown in Table 2 (found in “Appendix”), which corresponds to a gene cluster consisting of three gene orders of a known ABC transporter operon, encoding genes participating in a carbohydrate uptake system [33]. This example is illustrated in Fig. 1. In each of the gene orders shown in the figure, the product of gene number one is responsible for regulating the transcription of the other genes. The genes numbered two, three and four code for proteins that are part of the transporter machine. Gene number five codes for a protein that is responsible for some metabolism of the transported substrate, such as breaking down a complex sugar into smaller ones [34]. The genes numbered two, three, four and five are located close to each other in all gene orders and are included in a single operon, whose transcription is regulated by the product of gene number one [34].

Note that even though random shuffling by evolution forms different gene orders, not every order is conserved. In prokaryotic genomes, gene clusters often correspond to operons, which are adjacently localized genes that are co-transcribed and co-translated. Here, selection for specific gene orders in operons can be due to the assembly order of a multifunctional enzymatic complex [35] or the performance of consecutive reactions in a metabolic pathway [36]. The conserved gene order within operons also facilitates the evolution of multiple post-transcriptional and post-translational feedback mechanisms that regulate the expression of enzymes catalyzing different steps in the pathway according to metabolite availability [37]. Thus, the gene orders we see today are functionally conserved. In order to take this into consideration in our genome-rearrangement approach, we use a PQ-tree to represent the gene cluster and to model the possible evolutionary events that yielded the various gene orders (see Fig. 3).

**Fig. 2:**
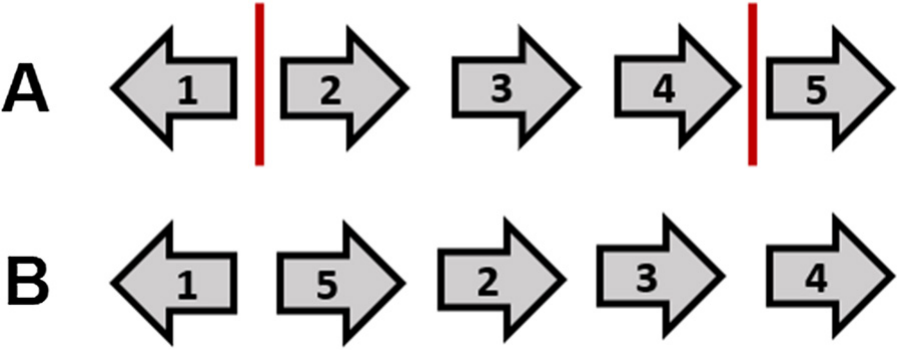
Computing the signed break-point distance between the first two gene orders from the example shown in Fig. 1. The two signed break-points are denoted by the red lines

**Fig. 3:**
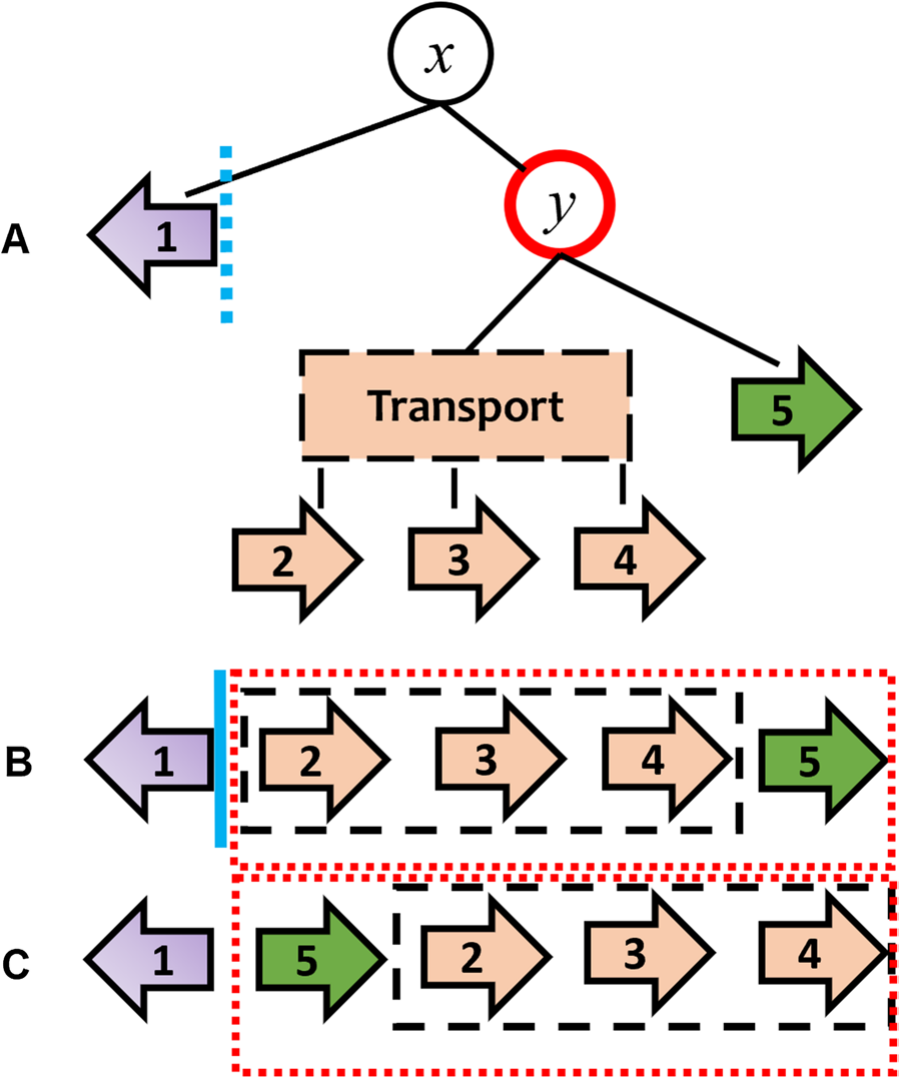
Re-considering the signed break-points (marked in blue) between the two gene orders exemplified in Fig. 2, this time taking into account the PQ-tree representing the corresponding gene cluster. **A** The PQ-tree. Note that in this figures, as well as the following figures, P-nodes are marked with circles and Q-nodes are marked with rectangles. **B** The first gene order. **C** The second gene order

Considering the first two gene orders in the cluster, the signed break-point distance between them is 2 (see Fig. 2). Now, let’s use our knowledge of the evolution of the gene cluster, using the PQ-tree (in Fig. 3), and reconsider the break-point between genes one and two. Looking at the PQ-tree, we can see that the proposed break-point actually falls between gene one and the P-node *y*, which represents genes from the cluster that are included in the same operon. Notice that the adjacency of gene one and P-node *y* appears both in the first gene order and in the second gene order. Therefore, according to the PQ-tree, this proposed break-point is functionally irrelevant and should not be penalized by the corresponding SIGR score! This makes biological sense, since gene 1 is a transcription factor that typically appears upstream to the operon it regulates, regardless of how the genes are arranged within the operon [34].

Next, consider a comparative analysis between these two gene orders based on a perfect reversals distance scenario. A perfect reversals scenario would acknowledge the observation that the transcription factor gene is not colinearly dependent on a specific gene from the transporter operon, however it would entail a long and expensive series of reversal operations: first, reversal of the sequence of genes 2–5, and then the individual reversal of each one of these genes. Although this may have been the actual evolutionary scenario that led to the second gene order, this scenario is quite uninformative in terms of both assessing the functional distance as well as interpreting the functional discrepency between the two gene orders. The point is, as would be observed by a PQ-tree guided block-interchange operation, that in the first order the sugar transporter is transcribed prior to the transcription of the sugar-metabolising gene, while in the second gene order the transcription order of these units is reversed.

## Previous related works

Permutations on strings representing syntenic gene blocks on genomes have been studied earlier by [38–42] and the idea of a maximal permutation pattern was introduced by Eres et al. [41]. In [8] an algorithm was proposed for representation and detection of gene clusters in multiple genomes using PQ-trees. The proposed algorithm identifies a minimal consensus PQ-tree, and it is proven that this tree is equivalent to a maximal permutation pattern and that each subgraph of the PQ-tree corresponds to a non-maximal permutation pattern. In that paper, the authors also present a general scheme to handle gene multiplicity and missing genes in permutations and give a linear time algorithm to construct the minimal consensus PQ-tree. These previous works fall in the domain of permutation discovery and PQ-tree construction. In contrast, the problems addressed in our paper take as input a previously constructed PQ-tree.

Three additional related works fall in the domain of “PQ-tree language distance computations”. The first problem is defined and solved in [7]. It asks, given a PQ-tree *T* and a string *s*, to find, among all permutations that *T* can generate, a permutation *p* such that the edit distances between *p* and *s* is minimal. Thus, the work of [7] used the input PQ-tree only as a constraint to guide the alignment process, and did not project the guiding information to the comparative score, as we do. Furthermore, the work of [7] did not consider genome rearangement operations at all, only string edit operations.

For break-point distance, two problems were proposed and solved in [43]. The first problem asks, given two PQ-trees over permutation gene orders, and a parameter *k*, whether there are two strings $S_1$ and $S_2$ generated from each of the trees, respectively, such that the break-point distance between $S_1$ and $S_2$ is up to *k*. The second problem asks, given a PQ-tree *T*, a set of *p* permutations and a parameter *k*, whether *T* can generate a permutation *s* such that the sum of the break-point distances between *s* and each of the given *p* permutations is bounded by *k*. However, in both of the problems addressed in [43], the order of the leaves in the input tree, as well as the actions taken on it are not taken into account. Additionally, the break-point distance is computed between two strings, and the tree is not a part of that distance computation. In this paper we employ a scoring strategy that takes the order of the leaves in the tree and the actions on the tree into account. In particular, we define the divergence from an ordered PQ-tree to a string by the rearrangement actions applied on the tree.

## Preliminaries

Let $S=s_1... s_n$ be a string. Denote by *S*[*i*] the character in position *i* in *S*, i.e. $S[i]=s_i$. In addition, denote by *S*[*i* : *j*] ($i \le j$) the subsequence of *S* from position *i* to position *j*, i.e. $S[i:j]=s_i... s_j$.

### PQ-tree—representing the pattern

A PQ-tree is a rooted tree with three types of nodes: *P-nodes*, *Q-nodes* and leaves. The children of a P-node can appear in any order, while the children of a Q-node must appear in either left-to-right order or right-to-left order. Booth and Lueker [5] were interested in permutations of a set, thus every member of *U* appears exactly once as a label of a leaf in the PQ-tree. We, on the other hand, allow each member of the set to appear as a label any non-negative number of times. The possible reordering of the children nodes in a PQ-tree can potentially create many equivalent PQ-trees. Booth and Lueker defined two PQ-trees *T* and $T'$ as *equivalent* (denoted $T \equiv T'$) if and only if one tree can be obtained by legally reordering the nodes of the other; namely, randomly permuting the children of a P-node, and reversing the children of a Q-node. A generalization of their definition, to allow for insertions and deletions, is defined as follows.

#### **Definition 1**

(*Quasi-Equivalence*) Two PQ-trees $T, \ T'$ are *quasi-equivalent* with parameter *d*, denoted by $T\cong _d T'$, if and only if $T'$ can be obtained by (a) randomly permuting the children of the P-nodes of *T*, (b) reversing the children of the Q-nodes of *T*, (c) deleting up to *d* leaves of *T*.

Denote by $T_x$ the subtree of a PQ-tree *T* rooted in the node *x*. Denote by $\textsf{Leaves}(x)$ the set of leaves of the PQ-tree $T_x$, $\textsf{span}(x)=|\textsf{Leaves}(x)|$ and for a set of nodes *U*, $\textsf{span}(U)=\sum _{v \in U} \textsf{span}(v)$. Denote by $\textsf{children}(x)$ the set of children of the node *x*, and let $root_T$ the root node of the tree *T*.

**Fig. 4:**
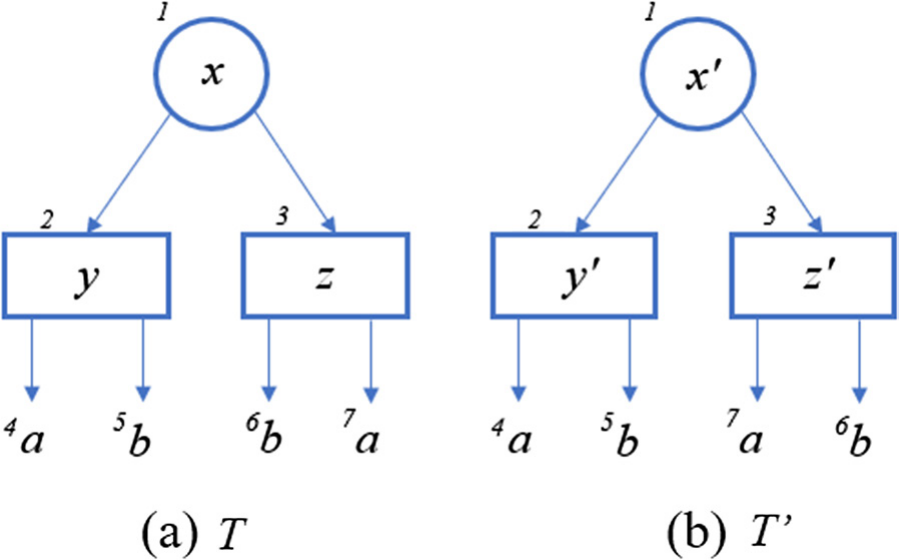
**a** PQ-tree *T*. **b** PQ-tree $T'$ which is quasi-equivalent to *T*. The colors are represented by the number assigned to each node, the labels of the leaves are the letters. Each internal node is *T* is marked by a letter (*x* for example), and its equivalent node is marked by the same letter with a dash ($x'$)

Given a PQ-tree *T*, we denote the label of a leaf *x* of *T* by $\textsf{label}(x)$. The *frontier* of the PQ-tree *T*, denoted *F*(*T*), is the sequence of labels on the leaves of *T* read from left to right. In addition to Definition 1, for each node in a PQ-tree (internal node or leaf), we define a unique “color” which will help us distinguish and map between nodes of two quasi-equivalent PQ-trees. Colors are used only for the analysis—they are not used explicitly in the algorithm. In the input we receive one PQ-tree (*T*) and “assign” arbitrary (but unique) colors to its nodes. In the algorithms, we perform actions on *T*, which reorder its nodes. We refer by $T'$ to the original tree (*T*) with reordered nodes. Thus, we use colors just to keep track of the nodes after the shuffling, see Fig. 4. From now on, when we say that two PQ-trees $T, \ T'$ are quasi-equivalent, we assume that the equivalence is with parameter *d*. In addition, we assume that the PQ-trees $T, \ T'$ are colored in the same unique colors (each color is assigned only to one node in *T* and at most one node in $T'$, and the nodes in $T'$ have the same colors as their corresponding nodes in *T*). In addition, we say that the *frontier* of $T'$, $F(T')$, is *derived* from *T*, and we call this a *derivation*. We can also say that $T'$ is ordered as $F(T')$. When a string *S* is derived from $T_x$, we also say that *S* is derived from *x*.

#### **Definition 2**

(*Equivalent Nodes*) Given two quasi-equivalent PQ-trees $T, \ T'$ with parameter *d* and two nodes $x \in T$ and $x' \in T'$, *x* and *y* are *equivalent nodes* if they share the same color.

In Fig. 4, the colors of the trees are shown as unique numbers near each node (notice that each node “keeps” its color in $T'$ compared to *T*). We say that the string $``abab$” is derived from *T*, because $``abab$” is the frontier of $T'$.

### Break-point distances

The definitions of our problems make use of the notion of break-point distance [43] to determine the distance between two strings, as defined below.

#### **Definition 3**

(*Gene Mapping*) Let $G=g_1,\ldots ,g_n$ and $H=h_1,\ldots ,h_m$ be two strings. A *gene mapping* of *G* and *H*, denoted by $\mathcal {M}$, is a set of pairs $(i,j) \in \{1,\ldots ,n\} \times \{1,\ldots ,m\}$ such that $g_i=h_j$^1^ and every position in *G* and *H* is in exactly one pair in $\mathcal {M}$. When no confusion arises, we suppose that the gene mapping contains the pairs of genes $(g_i,h_j)$ themselves.

#### **Definition 4**

(*Break-Point*) Given two strings $G=g_1,\ldots ,g_n$, $H=h_1,\ldots ,h_m$ and a gene mapping $\mathcal {M}$, a *break-point* between *G* and *H* is a pair of consecutive genes $g_ig_{i+1}$ in *G* (resp. $h_ih_{i+1}$ in *H*) such that the following is true: $g_i$ and $g_{i+1}$ (resp. $h_i$ and $h_{i+1}$) belong to $\mathcal {M}$, say, $(g_i,h_j),(g_{i+1},h_k) \in \mathcal {M}$ (resp. $(g_j,h_i),(g_k,h_{i+1}) \in \mathcal {M}$), but neither $k=j+1$ nor $k=j-1$.

Denote by $\mathsf {NUM_{BP}}(G,H,\mathcal {M})$ the number of break-points of *G* between *G* and *H* with respect to $\mathcal {M}$.

#### **Definition 5**

(*Break-Point Distance*) Let $S_1$ and $S_2$ be two strings. The *break-point distance* between $S_1$ and $S_2$, denoted by $\mathsf {d_{BP}}(S_1, S_2)$, is the minimum of $\mathsf {NUM_{BP}}(G,H,\mathcal {M})$ among all gene mappings $\mathcal {M}$ of *G* and *H*.

For example, suppose we are given the strings $S_1=abcd$ and $S_2=acbd$. Then, there exists exactly one gene mapping of $S_1$ and $S_2$. The break-points of $S_1$ are the pairs (*a*, *b*), (*c*, *d*). Therefore, the break-point distance between them is 2. We also can count the number of break-points of $S_2$, which are (*a*, *c*), (*b*, *d*).

We will use a variant of break-point distance that takes the signs of the characters in a string into account. Towards that, we define the notion of a signed string.

#### **Definition 6**

(*Signed String*) A *signed string* is a string where each character is assigned a sign (‘+’ or ‘−’).

When we say that a string *S* is a signed string, we suppose to have a sign function $\textsf{sign}{}$ that returns the sign of the character in each position in *S*. For the sake of illustration, in our examples, we will indicate the sign of each character by ‘+’ or ‘−’ of its left side.

#### **Definition 7**

(*Signed Break-Point*) Given two signed strings $G=g_1,\ldots ,g_n$, $H=h_1,\ldots ,h_m$ and a gene mapping $\mathcal {M}$, a *signed break-point* between *G* and *H* is a pair of consecutive genes $g_ig_{i+1}$ in *G* (resp. $h_jh_{j+1}$ in *H*) such that the following is true: $g_i$ and $g_{i+1}$ (resp. $h_i$ and $h_{i+1}$) belong to $\mathcal {M}$, say, $(g_i,h_j),(g_{i+1},h_k) \in \mathcal {M}$ (resp. $(h_i,g_j),(h_{i+1},g_k) \in \mathcal {M}$) but neither $k=j+1$, $\textsf{sign}{_G(i)}=\textsf{sign}{_H(j)}$ and $\textsf{sign}{_G(i+1)}=\textsf{sign}{_H(k)}$ nor $k=j-1$, $\textsf{sign}{_G(i)}=-\textsf{sign}{_H(j)}$ and $\textsf{sign}{_G(i+1)}=-\textsf{sign}{_H(k)}$.

Denote by $\mathsf {NUM_{SBP}}(G,H,\mathcal {M})$ the number of signed break-points of *G* between *G* and *H* with respect to $\mathcal {M}$.

#### **Definition 8**

(*Signed Break-Point Distance*) Let $S_1$ and $S_2$ be two signed strings. The *signed break-point distance* between $S_1$ and $S_2$, denoted by $\mathsf {d_{SBP}}(S_1,S_2)$, is the minimum of $\mathsf {NUM_{SBP}}(G,H,\mathcal {M})$ among all gene mappings $\mathcal {M}$.

For example, consider the signed strings $S_1=+a+b+c+d$ and $S_2=+a-b-d-c$. Then, there exists exactly one gene mapping of $S_1$ and $S_2$. The signed break-points of $S_1$ are the pairs (*a*, *b*), (*b*, *c*). Therefore, $\mathsf {d_{SBP}}(S_1,S_2)=2$. We can also count the number of signed break-points of $S_2$, which are (*a*, *b*), (*b*, *d*).

To accommodate deletions, we will use Definition 8 with respect to strings obtained from the given ones after deleting characters.

### Problem preliminaries

Given an internal node *x* in a PQ-tree *T*, we define its sign, $\textsf{sign}(x)$, as the majority sign of the leaves in $T_x$, $\textsf{Leaves}(x)$. If the number of negative signed leaves is equal to the number of positive signed leaves, then we abuse notation and consider $\textsf{sign}(x)$ as $+$ as well as −.

**Fig. 5:**
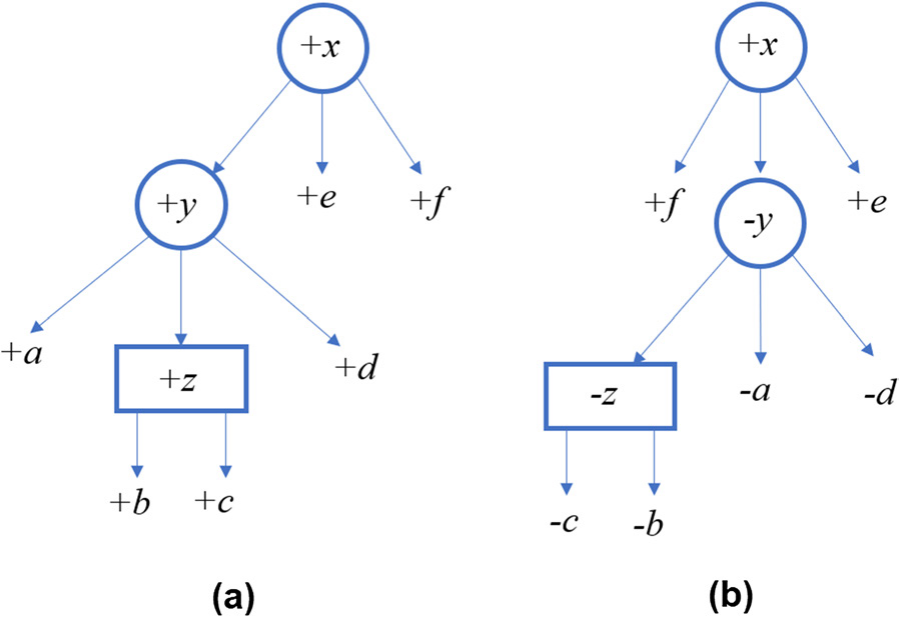
**a** A PQ-tree *T*. **b** A PQ-tree $T'$ that is equivalent to *T* and is ordered as *S*

Given a node *x* in a PQ-tree, let *S*(*x*) denote the signed string of colors of the nodes in $\textsf{children}(x)$ as they are ordered in the tree (from left to right). For example, consider the PQ-tree shown in Fig. 5a where the character assigned to each internal node is its color, then $S(z)=+b+c$, $S(y)=+a+z+d$ and $S(x)=+y+e+f$.

In order to measure the divergence from an ordered PQ-tree to a string, we take into account the actions performed on the PQ-tree to order it as needed. Towards defining the divergence from an (ordered) PQ-tree *T* to a string *S*, we first define a penalty for taking an action on an internal node of a PQ-tree, denoted by $\Delta _{\textsf{violation}}$. The penalty $\Delta _{\textsf{violation}}$ is a combination of several types of penalties. The first type concerns cases where large units “jump” while reordering the children of a P-node *x* to have the same order as the children of its equivalent node $x'$. Specifically, we want to penalize according to the sizes of these units: We will not penalize a single leaf that jumps, but only units whose size is larger than 1, and the penalty for these units increases with their sizes. Thus, we consider the following penalty. If the size of a unit that jumps is *t*, then we penalize this operation by $(t-1)/2$. In particular, a leaf does not get penalized. To do so, we build a graph $G(x,x')$ (defined formally later), whose vertex set is the children of the P-node. Each vertex has a weight relative (as mentioned before and will defined ahead) to its size (defined as the span of the child it represents). Roughly speaking, the graph *G* has an edge for each pair of children that “changed their order” (with respect to the their signs). To be precise, we need the following definition.

#### **Definition 9**

(*Change Of Signed Order*) Let $x, x'$ be two equivalent P-nodes of two quasi-equivalent PQ-trees *T* and $T'$ with parameter *d*, $S(x)=c_1,\ldots ,c_n$ be the signed string of colors of the nodes in $\textsf{children}(x)$ that were not deleted, as they ordered in *T*, $S(x')=c'_1,\ldots ,c'_{n'}$ be the signed string of colors of the nodes in $\textsf{children}(x')$, as they ordered in $T'$, and $\mathcal {M}$ be the^2^ gene mapping of *S*(*x*) and $S(x')$. Given two nodes *y* (with color $c_i$ and $y'$ is the equivalent node of *y* in $T'$) and *z* (with color $c_j$ and $z'$ is the equivalent node of *z* in $T'$) in $\textsf{children}(x)$, say, $j>i$, and such that $(c_i,c'_k),(c_j,c'_t) \in \mathcal {M}$ for some $c'_k$ and $c'_t$, *y* and *z* *change their signed order* if the following are both false.

- $$t>k$$, $$\textsf{sign}(y)=\textsf{sign}(y')$$ and $$\textsf{sign}(z)=\textsf{sign}(z')$$.
- $$t<k$$, $$\textsf{sign}(y)=-\textsf{sign}(y')$$ and $$\textsf{sign}(z)=-\textsf{sign}(z')$$.

We denote by $G[x,x']$ the graph (described before Definition 9) of two equivalent P-nodes *x* and $x'$ of two quasi-equivalent PQ-trees. Formally, it is defined as follows.

#### **Definition 10**

(${G[x,x']}$) Given two equivalent P-nodes $x, x'$ of two quasi-equivalent PQ-trees with parameter *d*, $G[x,x'] = (V,E,w)$ is the undirected graph with vertex set *V*, edge set *E* and vertex weight function *w* which defined as follows.

- $$V=\textsf{children}(x)$$.
- $$E=\{(v,u)|u \ and \ v \ changed \ their \ signed \ order\}$$.
- For each $$x \in V, \ w(x)=(\textsf{span}(x)-1)/2$$.

Notice that the graph is dependent on the colors, to determine the edge set.

After building $G[x,x']$, we find the minimum weight of a vertex cover of $G[x,x']$ in order to sum all penalties for the units that jumped while reordering the children of a P-node. Observe that by computing the minimum weight of a vertex cover, we identify a “best” (in terms of penalty) set of nodes that jump.

#### **Definition 11**

(*Minimum Weighted Vertex Cover*) Let $G=(V,E,w)$ be a graph with vertex set *V*, edge set *E* and vertex weight function *w*. The *minimum weighted vertex cover* of *G* is the minimum weight^3^ among all vertex covers of *G*.^4^

#### **Definition 12**

($\Delta ^P_{\textsf{jump}}(x,x')$) Given two equivalent P-nodes *x* and $x'$ of two quasi-equivalent PQ-trees with parameter *d*, and the weight *t* of a minimum weighted vertex cover of $G[x,x']$, the *jump violation* between *x* and $x'$, denoted by $\Delta ^P_{\textsf{jump}}(x,x')$, is *t*.

Now, we define a violation between two equivalent internal nodes. Towards that, given two equivalent nodes $x,x'$ of two quasi-equivalent PQ-trees *T* and $T'$ (where *x* is not deleted), let $\textsf{isFlipped}(x,x')$ be a procedure that returns 1 if *x* and $x'$ “flipped”, and 0 otherwise. That is, if *x* and $x'$ are leaves, $\textsf{isFlipped}(x,x')=1$ if $\textsf{sign}(x)=-\textsf{sign}(x')$; otherwise, $\textsf{isFlipped}(x,x')=0$. If *x* and $x'$ are internal nodes, $\textsf{isFlipped}(x,x')=1$ if for each child $y \in \textsf{children}(x)$ that is not deleted, $\textsf{isFlipped}(y,y')=1$ where $y'$ is the child of $x'$ that is equivalent node of *y*, and the order of $\textsf{children}(x')$ in $T'$ is the reversal of the order of $\textsf{children}(x)$ in *T*; otherwise, $\textsf{isFlipped}(x,x')=0$.

Given an internal node *x*, denote by $\tilde{\mathcal {S}}_d(x)$ the set of signed strings where $\tilde{S} \in \tilde{\mathcal {S}}_d(x)$ if $\tilde{S}$ is obtained from *S*(*x*) by deleting up to *d* leaves from $T_x$.

#### **Definition 13**

($\Delta _{\textsf{violation}}^d(x,x')$) Given two equivalent internal nodes *x* and $x'$ of two quasi-equivalent PQ-trees with parameter *d*, and input numbers $\delta ^Q_{\textsf{ord}}$ and $\delta ^Q_{\textsf{flip}}$, the *violation* between *x* and $x'$, denoted by $\Delta _{\textsf{violation}}^d(x,x')$, is defined as follows.

- If *x* is a P-node, $\Delta ^{\textsf{P}}_{\textsf{violation}}(x,x') =\displaystyle \min _{\tilde{S} \in \tilde{\mathcal {S}}_d(x)} \mathsf {d_{SBP}}(\tilde{S}, S(x')) + \Delta ^P_{\textsf{jump}}(x,x')$.
- If *x* is a Q-node, $\Delta ^{\textsf{Q}}_{\textsf{violation}}(x,x') = \delta ^Q_{\textsf{ord}}{}\cdot \displaystyle \min _{\tilde{S} \in \tilde{\mathcal {S}}_d(x)} \mathsf {d_{SBP}}(\tilde{S}, S(x')) + \textsf{isFlipped}(x,x')\cdot \delta ^Q_{\textsf{flip}}$.

When we use the notation $\Delta _{\textsf{violation}}^d$, if *d* is clear from the context, we will use the notation $\Delta _{\textsf{violation}}$ instead. In Definition 13, one of the penalties for reordering the children of a Q-node is the flip penalty ($\delta ^Q_{\textsf{flip}}$), and it is performed if the Q-node has flipped (as indicated by $\textsf{isFlipped}$). When considering this event, notice the case where a Q-node *x* flipped and all of the non-deleted children in $\textsf{children}(x)$ flipped as well (including the P-nodes). In this case, we penalise each of the non-deleted children in $\textsf{children}(x)$ and *x* as well for flipping, but, in fact, the event is flipping only *x*. Thus, we unnecessarily penalise the non-deleted children in $\textsf{children}(x)$. Therefore, we employ a flip correction procedure as defined next.

#### **Definition 14**

($\textsf{FlipCorrection}(x,x')$) Given two equivalent internal nodes *x* and $x'$ of two quasi-equivalent PQ-trees with parameter *d*, the *flip correction* between them, denoted by $\textsf{FlipCorrection}(x,x')$, is 0 if not all of $\textsf{children}(x)$ flipped or deleted, and otherwise it is the sum of the flip penalties of all of the Q-nodes and leaves in $\textsf{children}(x)$ that were not deleted.

Finally, after defining the violation between equivalent nodes, we simply sum up the violations (and corrections) of all internal nodes to define the divergence from an ordered and labeled PQ-tree to a signed string as follows.

#### **Definition 15**

($\textsf{Diverge}_{d_T,d_S}(T,S)$) Let *T* be an (ordered and leaf-labeled) PQ-tree, and *S* be a signed string. Let $\mathcal {O}_{T}$ be the set of all quasi-equivalent $T'$ PQ-trees of *T* with parameter $d_T$ (i.e. $T\cong _{d_T} T'$) such that $T'$ is ordered as a subsequence of *S* obtained by deleting up to $d_S$ characters from *S*. For any $T' \in \mathcal {O}_{T}$, let $\mathcal {M}_{T'}$ be the unique^5^ mapping that maps each node in $T'$ to its equivalent node in *T*. Then,

$$\begin{aligned} \textsf{Diverge}_{d_T,d_S}(T,S) = \min _{T' \in \mathcal {O}_{T}} \sum _{(x,x') \in \mathcal {M}_{T'}} (\Delta _{\textsf{violation}}(x,x') - \textsf{FlipCorrection}(x,x')). \end{aligned}$$

In case $d_T=0$ and $d_S=0$, we will use the notation $\textsf{Diverge}(T,S)$ instead of $\textsf{Diverge}_{d_T,d_S}(T,S)$ for simplicity.

For example, consider the ordered PQ-tree *T* in Fig. 5, which is ordered as $+a+b+c+d+e+f$, and the signed string $S=+f-c-b-a-d+e$. To order *T* as *S*, we flip the Q-node *z*, and for this we pay $\Delta ^{\textsf{Q}}_{\textsf{violation}}(z,z') = \delta ^Q_{\textsf{flip}}$. Now, consider the P-node *y*. We swap between *a* and *z*, so $\mathsf {d_{SBP}}(S(y),S(y'))=1$, and hence $\Delta ^{\textsf{P}}_{\textsf{violation}}(y,y') = 1$ (notice that there is no jump of a large unit, because the leaf *a* can jump). Finally, for the P-node *x*, $\Delta ^P_{\textsf{jump}}(x,x')=0$ (we can order the children by moving the leaf *f* to the left-most position). In addition, $\mathsf {d_{SBP}}(S(x), S(x'))=1$. Thus, for *x* we pay $\Delta ^{\textsf{P}}_{\textsf{violation}}(x,x') = 1$. In total $\textsf{Diverge}(T,S)=\Delta ^{\textsf{Q}}_{\textsf{violation}}(z,z')+\Delta ^{\textsf{P}}_{\textsf{violation}}(y,y')+\Delta ^{\textsf{P}}_{\textsf{violation}}(x,x') = \delta ^Q_{\textsf{flip}}+2$.

### Problem definitions

#### $\mathsf {Constrained \ TreeToString \ Divergence}$

The input to $\textsf{CTTSD}{}$ consists of two signed permutations of length *n*, $S_1=\sigma _1 \cdots \sigma _n \in \Sigma ^n$, $|\Sigma |=n$, such that $\sigma _i \ne \sigma _j$ for all $1 \le i<j \le n$, and $S_2=\lambda _1 \cdots \lambda _n \in \Sigma ^n$ such that $\lambda _i \ne \lambda _j$ for all $1 \le i<j \le n$; a PQ-tree *T* ordered as $S_1$ with $m_p$ P-nodes and $m_q$ Q-nodes; and two numbers $\delta ^Q_{\textsf{ord}}$ and $\delta ^Q_{\textsf{flip}}$. We aim to perform actions on *T* to reorder it as $S_2$. That is, we reorder *T* as $T'$ so that $F(T')=S_2$. If this is not possible, we answer “NO”. Else, we return the divergence from *T* to $S_2$. Concretely, $\textsf{CTTSD}{}$ is defined as follows.

##### **Definition 16**

($\mathsf {Constrained \ TreeToString \ Divergence}$) Given two signed permutations $S_1$ and $S_2$ of the same length and the same characters, two numbers $\delta ^Q_{\textsf{ord}}$ and $\delta ^Q_{\textsf{flip}}$, and a PQ-tree *T* ordered as $S_1$, return $\textsf{Diverge}(T,S_2)$ or answer “NO” if $S_2$ cannot be derived from *T*.

In “Constrained TreeToString Divergence: algorithm” section, we propose an algorithm to solve this problem.

##### $\mathsf {TreeToString \ Divergence}$

Generalizing $\textsf{CTTSD}{}$, in $\textsf{TTSD}{}$ we do not assume that the input strings are permutations, and we allow deletions. The input to $\textsf{TTSD}{}$ consists of two signed strings, $S_1=\sigma _1 \cdots \sigma _m \in {\Sigma ^m_T}$ and $S_2=\lambda _1 \cdots \lambda _n \in {\Sigma ^n_S}$; a PQ-tree *T* ordered as $S_1$ with $m_p$ P-nodes and $m_q$ Q-nodes; $d_T\in \mathbb {N} \cup \{0\}$, which specifies the number of allowed deletions from *T*; $d_S\in \mathbb {N}\cup \{0\}$, which specifies the number of allowed deletions from $S_2$; and two numbers $\delta ^Q_{\textsf{ord}}$, $\delta ^Q_{\textsf{flip}}$ indicating the penalty of the events of changing order and flipping, respectively, a Q-node. In $\textsf{TTSD}{}$ we perform actions on *T* to reorder it as a subsequence $S_2'$ of $S_2$, allowing $d_T$ deletions from *T*, and so that $S_2'$ is obtained from $S_2$ by using up to $d_S$ deletions from $S_2$. That is, after reordering *T* as $T'$ (and performing up to $d_T$ deletions), $F(T')=S_2'$. If it is not possible, we answer “NO”. Else, we return the divergence from *T* to $S_2'$ corresponding to $d_T$ and $d_S$. Concretely, $\textsf{TTSD}{}$ is defined as follows.

###### **Definition 17**

($\mathsf {TreeToString \ Divergence}$) Given two signed strings $S_1$ and $S_2$, a PQ-tree *T* ordered as $S_1$, and parameters $d_T$, $d_S$, $\delta ^Q_{\textsf{ord}}$, $\delta ^Q_{\textsf{flip}}$, return $\textsf{Diverge}_{d_T,d_S}(T,S_2)$ or “NO” if $S_2$ cannot be derived from *T* with respect to $d_T$ and $d_S$.

### Parameterized complexity

Let $\Pi$ be an NP-hard problem. In the framework of Parameterized Complexity, each instance of $\Pi$ is associated with a *parameter* *k*. Here, the goal is to confine the combinatorial explosion in the running time of an algorithm for $\Pi$ to depend only on *k*. Formally, we say that $\Pi$ is *fixed-parameter tractable (*FPT*)* if any instance (*I*, *k*) of $\Pi$ is solvable in time $f(k)\cdot |I|^{\mathcal {O}(1)}$, where *f* is an arbitrary function of *k*.

### Algorithms preliminaries

Given a node *x* and the numbers of deletions $k_T$ and $k_S$ of a derivation, the length of the derived string $S'$ can be calculated using the length function given in Definition 18 below.

#### **Definition 18**

(*The Length Function*) $L(x,k_T,k_S) \doteq \textsf{span}(x) - k_T + k_S$.

Using the length function and the start point *s* of the derivation, the end-point of the derivation can be calculated using the end-point function given in Definition 19 below.

#### **Definition 19**

(*The End-Point Function*) $E(x,s,k_T,k_S) \doteq s-1+L(x,k_T,k_S)$.

Let $\mathcal {A}{}$ be a DP table used in an algorithm. Addressing $\mathcal {A}{}$ with some of its indices given as dots refers to the subtable of $\mathcal {A}{}$ that is comprised of all entries of $\mathcal {A}{}$ indexed by the indices that are specified (i.e., all indices not marked by dots). For example, $\mathcal {A}[x,\cdot ,\cdot ,\cdot ,\cdot ]$, refers to the subtable of $\mathcal {A}{}$ that is comprised of all entries of $\mathcal {A}{}$ whose first entry is *x*.

In the algorithms, for a given node *x* in a PQ-tree *T*, number of positive signed leaves in $T_x$, *pos*, a possible sign $s \in \{+,-\}$, and the number of deletions from $T_x$, $k_T$ (if not implied by context, then $k_T=0$), we say that *pos* is *consistent* with *s* (or *s* is *consistent* with *pos*) if $s=+$ and $pos \ge (\textsf{span}(x)-k_T)/2$ or if $s=-$ and $pos \le (\textsf{span}(x)-k_T)/2$. Moreover, in some situations, for a given set of nodes *C*, we will sometimes use a set $N \subseteq C$ to describe the nodes in *C* that have a negative sign: for a given node $x \in C$, if $x \in N$, then $\textsf{sign}(x)=-$; otherwise, $\textsf{sign}(x)=+$. In these situations, we say that *pos* is *consistent* with *N* (or *N* is *consistent* with *pos*) if $x \in N$ and $pos \le (\textsf{span}(x)-k_T)/2$ or if $x \notin N$ and $pos \ge (\textsf{span}(x)-k_T)/2$.

## $\mathsf {Constrained \ TreeToString \ Divergence}$: algorithm

In this section, we present a greedy algorithm to solve $\textsf{CTTSD}{}$. Our algorithm consists of three components: the main algorithm, and two procedures called P-Mapping and Q-Mapping. We first present and explain the main algorithm and the procedures. Afterwards, we demonstrate the execution of the algorithm and analyze its running time.

### The main algorithm

Recall that the input to $\textsf{CTTSD}{}$ consists of two signed permutations $S_1$ and $S_2$ of length *n*, two numbers $\delta ^Q_{\textsf{ord}}$ and $\delta ^Q_{\textsf{flip}}$, and a PQ-tree *T* ordered as $S_1$. If $S_2$ can be derived from *T*, then the output of the algorithm is the divergence from *T* to $S_2$, $\textsf{Diverge}(T,S_2)$. Otherwise, the output is “NO” (specifically, the algorithm returns $\infty$).

The main algorithm (whose pseudo-code is given in Algorithm 1) constructs a 2-dimensional DP table $\mathcal {A}{}$ of size $m' \times n$ where $m'=n + m_p + m_q$ is the number of nodes in *T*. For each node *x* in *T* and index $\ell$, $\mathcal {A}$ has an entry $\mathcal {A}[x,\ell ]$. In the algorithm, for each node *x*, we keep two indices $\ell$ and *r* (denoted by $x.\ell$ and *x*.*r* respectively) such that $S_2[x.\ell :x.r]$ is derived from $T_x$. Then, the purpose of an entry of the DP table, $\mathcal {A}[x,x.\ell ]$, is to hold the divergence from the subtree $T_x$ to the subsequence $S_2[x.\ell :x.r]$ of $S_2$. That is,

$$\begin{aligned} \mathcal {A}{[x,x.\ell ]} = \textsf{Diverge}(T_x, S_2[x.\ell :x.r]). \end{aligned}$$

If any subsequence of $S_2$ starting at position $\ell$ cannot be derived from $T_x$, then $\mathcal {A}[x,\ell ] = \infty$.

Some entries of the DP table define illegal derivations. Such are derivations where the length of the frontier of the subtree is larger than the length of the longest subsrting starting at the specified index $\ell$. These entries are called *invalid entries* and their value is defined as $\infty$ throughout the algorithm. Formally, an entry $\mathcal {A}[x,\ell ]$ is invalid if $\textsf{span}(x)>n-\ell +1$.

The algorithm first initializes the entries of $\mathcal {A}{}$ that are meant to hold divergences of derivations of every possible subsequence of $S_2$ (a single character) from the leaves of *T*. Specifically, for a leaf *x*, if it did not flip, we put 0 in the corresponding entry. If *x* did flip, we put $\delta ^Q_{\textsf{flip}}$. After that, we update $x.\ell$ and *x*.*r*. As described in the initialization, if $\textsf{label}(x) = S_2[\ell ]$, $S_2[\ell ]$ ($S_2[\ell :\ell ]$) is derived from $\textsf{label}(x) = S_2[\ell ]$, then we put 0 or $\delta ^Q_{\textsf{flip}}$ in $\mathcal {A}{[x,\ell ]}$ and we put $\ell$ in $x.\ell$ and *x*.*r*.

After the initialization, all other entries of $\mathcal {A}{}$ are filled as follows. Go over the internal nodes of *T* in postorder. For every internal node *x*, go in descending order over every index $1 \le \ell \le n$ that can be a start index for the subsequence of $S_2$ derived from $T_x$ (in case of invalid entry, we continue to the next iteration). For every *x* and $\ell$, use the algorithm for P-mapping or Q-mapping according to the type of *x*. Both algorithms receive the following input: the node *x*, $S_2$, start and end indices $\ell ,e$ of a subsequence of $S_2$, and the collection of derivations of the children of *x* (entries of $\mathcal {A}{}$ that have already been computed and hold the divergence of a derivation). In addition, the Q-Mapping algorithm receives as input the penalty parameters $\delta ^Q_{\textsf{ord}}$ and $\delta ^Q_{\textsf{flip}}$. After being called, both algorithms return the divergence from $T_x$ to $S_2[\ell :e]$, that is, $\textsf{Diverge}(T_x, S_2[\ell :e])$.
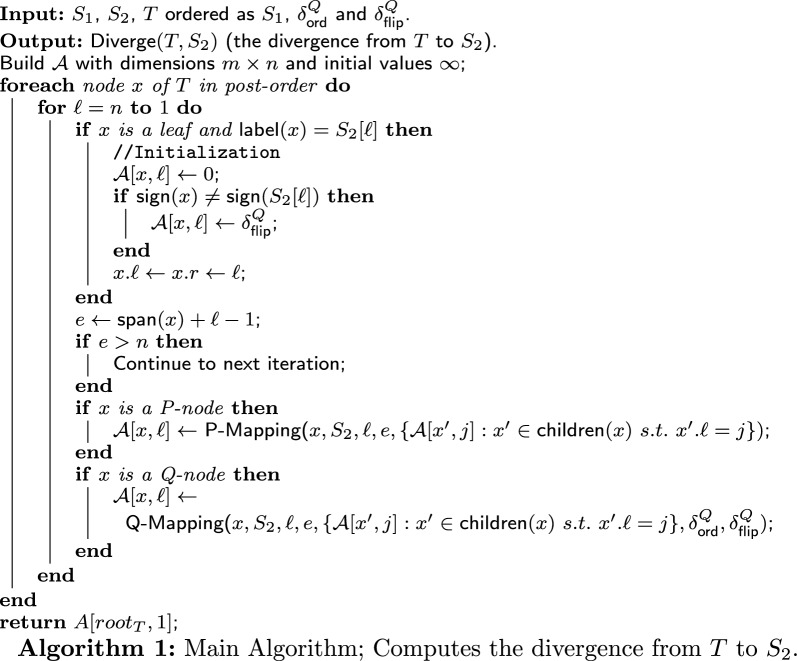


Finally, having filled the DP table, $\mathcal {A}{[root_T,1]}$ holds the divergence from *T* to $S_2$ ($\textsf{Diverge}(T, S_2)$), and so we return $\mathcal {A}{[root_T,1]}$.

### P-node mapping: the algorithm

Recall that the input consists of a P-node *x*, a string $S_2$, two indices $\ell$ and *e*, and a set of derivations $\mathcal {D}{}$. Notice that each value in $\mathcal {D}{}$ is the divergence from the subtree rooted in a child *c* of *x* to $S_2[c.\ell :c.e]$, where $S_2[c.\ell :c.e]$ is a subsequence of $S_2[\ell :e]$ that is derived from $T_c$. These values, $\mathcal {A}[c,c.\ell ]$ for each $c \in \textsf{children}(x)$, were calculated in earlier iterations and saved in $\mathcal {D}{}$. If $S_2[\ell :e]$ can be derived from $T_x$, then the output of the algorithm is the divergence from $T_x$ to $S_2[\ell :e]$, $\textsf{Diverge}(T_x,S_2[\ell :e])$. Otherwise, the output is “NO” (specifically, the algorithm returns $\infty$). Denote by $T'_x$ the quasi-equivalent PQ-tree of $T_x$ ordered as $S_2[\ell :e]$. Note that if $T'_x$ exists, then it is unique (because we deal with permutations and forbid deletions).

The algorithm (whose pseudo-code is given in Algorithm 2) first checks if the interval $[\ell ,e]$ can be “completed” by all of the intervals defined by the indices of the children of *x*. Specifically, we check if there is any order of the children of *x*, say, ordered as $c_1,\dots ,c_{|\textsf{children}(x)|} \in \textsf{children}(x)$, such that $c_1.\ell = \ell$, $c_{|\textsf{children}(x)|}.e = e$, and for each $1 \le j \le |\textsf{children}(x)|-1$, $c_j.e + 1 = c_{j+1}.\ell$. If there is no such order, then the interval $[\ell ,e]$ cannot be completed, and so $S_2[\ell ,e]$ cannot be derived from $T_x$. In this case, we return $\infty$. Otherwise, $S_2[\ell :e]$ can be derived from $T_x$ by reordering the children of *x* according to the unique order that completes the interval $[\ell ,e]$. Second, we sum up all of the values in $\mathcal {D}{}$ (and store the sum in the variable *childrenDist*). Next, we calculate the violation between *x* and its equivalent node $x'$ in $T'_x$, $\Delta ^{\textsf{P}}_{\textsf{violation}}(x,x')$, according to Definition 13 (and store the result in the variable *violation*). Finally, we return the sum of *childrenDist* and *violation*, which is the divergence from $T_x$ to $S_2[\ell :e]$, $\textsf{Diverge}(x,S_2[\ell :e])$ (according to Definition 15).
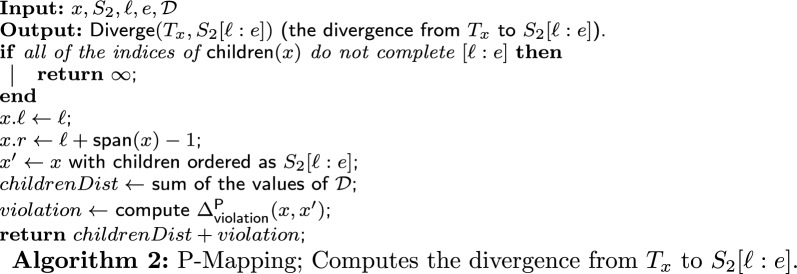


### Q-node mapping: the algorithm

Recall that the input consists of a Q-node *x*, a string $S_2$, two indices $\ell$ and *e*, a set of derivations $\mathcal {D}{}$ and penalty parameters $\delta ^Q_{\textsf{ord}}$ and $\delta ^Q_{\textsf{flip}}$. Notice that each value in $\mathcal {D}{}$ is the divergence from the subtree rooted in a child *c* of *x* to $S_2[c.\ell :c.e]$, where $S_2[c.\ell :c.e]$ is a subsequence of $S_2[\ell :e]$ and is derived from $T_c$. These values, $\mathcal {A}[c,c.\ell ]$ for each $c \in \textsf{children}(x)$, were calculated in earlier iterations and saved in $\mathcal {D}{}$. If $S_2[\ell :e]$ can be derived from $T_x$, then the output of the algorithm is the divergence from $T_x$ to $S_2[\ell :e]$, $\textsf{Diverge}(T_x,S_2[\ell :e])$. Otherwise, the output is “NO” (specifically, the algorithm returns $\infty$). Denote by $T'_x$ the (unique) quasi-equivalent PQ-tree of $T_x$ ordered as $S_2[\ell :e]$.

The algorithm (whose pseudo-code is given in Algorithm 3) first checks if the interval $[\ell ,e]$ can be “completed consecutively” by all of the intervals defined by the indices of the children of *x*. Specifically, we check if there is a consecutive order of the children of *x*, say, ordered as $c_1,\dots ,c_{|\textsf{children}(x)|} \in \textsf{children}(x)$, such that $c_1.\ell = \ell$, $c_{|\textsf{children}(x)|}.e = e$, and for each $1 \le j \le |\textsf{children}(x)|-1$, $c_j.e + 1 = c_{j+1}.\ell$. As apposed to a P-node, here the order of the children completing the interval $[\ell ,e]$ must be consecutive with respect to their indices (the same order as the children of *x* in *T* or the reverse order). If there is no such order, then the interval $[\ell ,e]$ cannot be completed consecutively, and so $S_2[\ell :e]$ cannot be derived from $T_x$. In this case, we return $\infty$. Otherwise, $S_2[\ell :e]$ can be derived from $T_x$ by keeping the order of the children of *x*, or flipping it. Second, we sum up all of the values in $\mathcal {D}{}$ (and store the sum in the variable *childrenDist*). Next, we calculate the violation between *x* and its equivalent node $x'$ in $T'_x$, $\Delta ^{\textsf{Q}}_{\textsf{violation}}(x,x')$, according to Definition 13 (and store the result in the variable *violation*). Afterwards, we calculate the flip correction according to Definition 14 (and store the result in the variable *childrenFlipCorrection*). Finally, we return the sum of *childrenDist* and *violation* minus *childrenFlipCorrection*, which is the divergence from $T_x$ to $S_2[\ell :e]$, $\textsf{Diverge}(x,S_2[\ell :e])$ (according to Definition 15). 
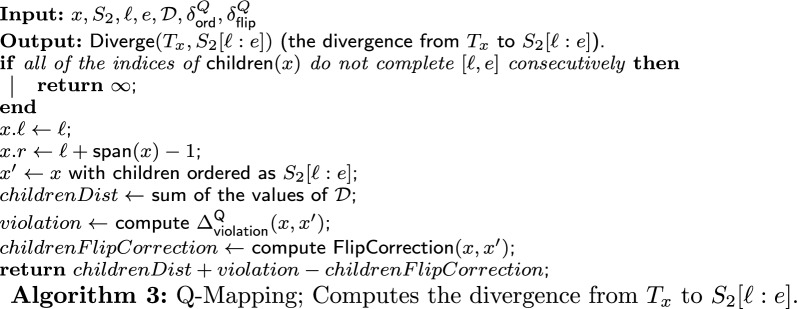


### Example

Consider the following input: $S_1=+a+b+c+d+e+f$, $S_2=+f-c-b-a-d-e$, PQ-tree T ordered as $S_1$, $\delta ^Q_{\textsf{ord}}=3$ and $\delta ^Q_{\textsf{flip}}=3$. We iterate through the nodes of the tree in post-order, thus we initiate the leaves before their parents. For each leaf $x \in \textsf{Leaves}(root_T)$, if $\textsf{label}{(x)} = S_2[\ell ]$, then $\mathcal {A}(x,\ell )=0$; otherwise, $\mathcal {A}(x,\ell ) = \infty$.

**Fig. 6:**
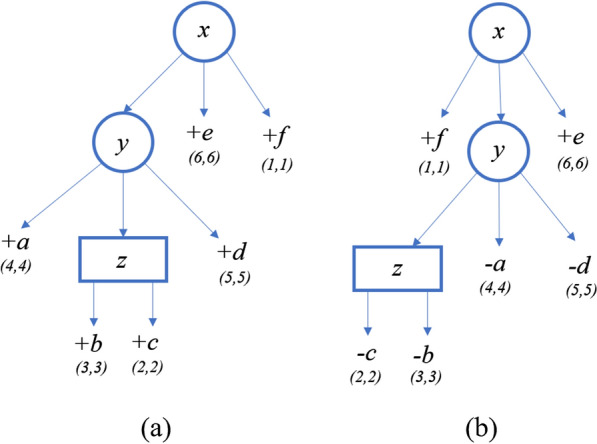
**a** PQ-tree *T*. **b** PQ-tree $T'$, which is quasi-equivalent to *T* and ordered as $S_2$

Figure 6 describes the PQ-tree *T* and its quasi-equivalent PQ-tree $T'$ ordered as $S_2$, after the initialization of the leaves only. Notice that the order of the initialization is in fact different, in postorder; for simplicity, we show the tree where only the leaves are initialized. In addition, in the figures, the equivalent nodes of *T* and $T'$ are shown as the same nodes. But in the explanations, in order to distinguish between them, for each node $x \in T'$, we denote it by $x'$. The pair of numbers shown in the figure near a node represent its $\ell$ and *r* values. In addition, the sign $+$ or − near a node represents its sign. For example, for $a \in T$, $a.\ell =a.r=4$ and $\textsf{sign}(a) = +$. For each internal node, the character assigned to it represents its color.

**Fig. 7:**
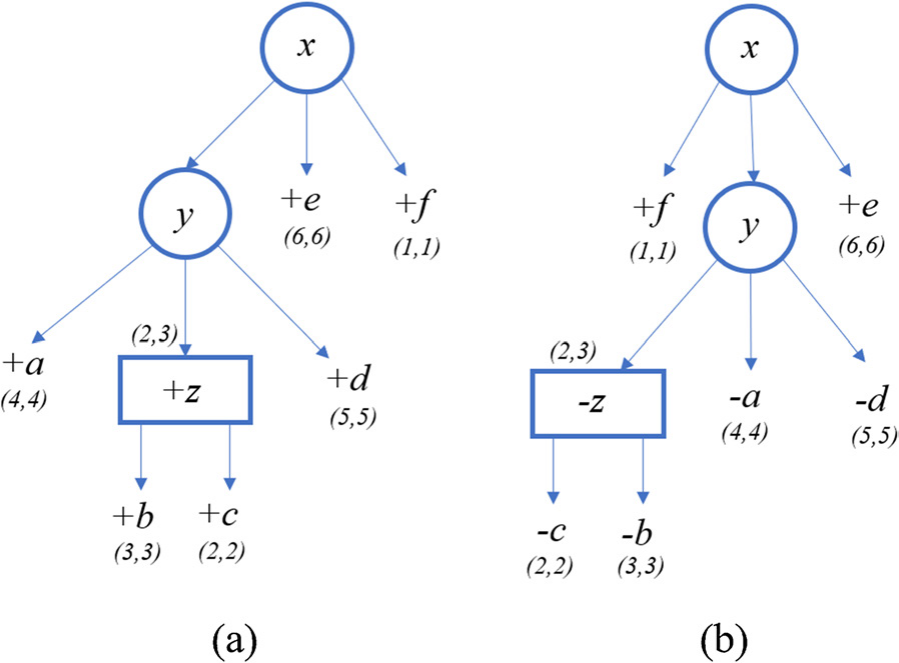
**a** PQ-tree *T*. **b** PQ-tree $T'$, which is quasi-equivalent to *T* and ordered as $S_2$

First, consider the iteration where $\mathcal {A}[z,2]$ is calculated (the values for all other entries with node *z* is $\infty$). The intervals of the children of *z*, [3, 3] and [2, 2], complete the interval (2, *e*) consecutively where $e=2+\textsf{span}(z)-1=3$. Thus, the subsequence $S_2[2:3]$ is derived from $T_z$, and in order to generate it we need to flip the order of $\textsf{children} \,(z)$. For this the penalty of flipping, $\delta ^Q_{\textsf{flip}}$, is applied, and so $\Delta ^{\textsf{Q}}_{\textsf{violation}}(z,z')=\delta ^Q_{\textsf{flip}}=3$. $childrenDist=\mathcal {A}[b,3]+\mathcal {A}[c,2]=6$ because $\mathcal {A}[b,3]=\mathcal {A}[c,2]=3$. $\textsf{FlipCorrection}(z,z')=6$ because both *b* and *c* have been flipped. Therefore, $\mathcal {A}[z,2]=childrenDist + violation - childrenFlipCorrection=3$. Now, we update *z*’s indices, $z.\ell =2$ and $z.r=3$. Figure 7 describes the PQ-tree *T* and its quasi-equivalent PQ-tree $T'$ after calculating $\mathcal {A}[z,2]$.

**Fig. 8:**
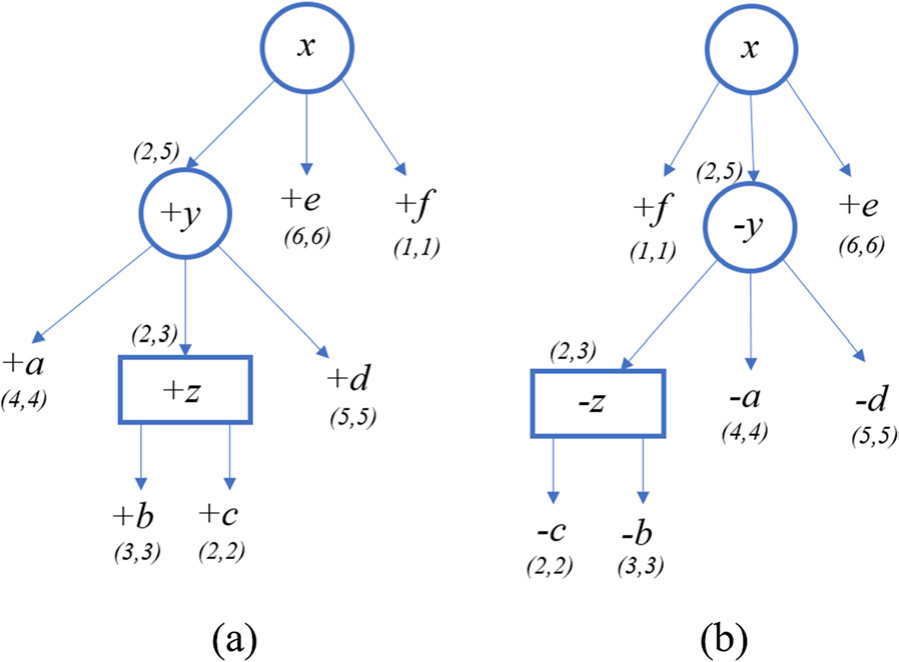
**a** PQ-tree *T*. **b** PQ-tree $T'$, which is quasi-equivalent to *T* and ordered as $S_2$

Next, consider the iteration where $\mathcal {A}[y,2]$ is calculated (the values for all other entries with node *y* is $\infty$). Recall that Fig. 7 describes *T* and $T'$ after calculating the entries of the children of the P-node *y*. The intervals of the children complete the interval (2, *e*), where $e=2+\textsf{span}(y)-1=5$, so we can continue with the iteration. $childrenDist=\mathcal {A}[a,4]+\mathcal {A}[z,2]+\mathcal {A}[d,5]=9$. $\mathsf {d_{SBP}}(S(y), S(y'))=1$ where the pair (*z*, *d*) is the only singed break-point (note that $S(y)=+a+z+d$, $S(y')=-z-a-d$). $\Delta ^P_{\textsf{jump}}(y,y')=0$ because to reorder the children of *y* as $S_2[2:5]$ we can move *z* only (and its size is 1). Therefore, $\Delta ^{\textsf{P}}_{\textsf{violation}}(y,y') = \mathsf {d_{SBP}}(S(y), S(y')) + \Delta ^P_{\textsf{jump}}(y,y')=1$. Then, $\mathcal {A}[y,2]=10$ and we can update *y*’s indices, $y.\ell =2$ and $y.r=5$. Figure 8 describes the PQ-tree *T* and its quasi-equivalent PQ-tree $T'$ after calculating $\mathcal {A}[y,2]$.

**Fig. 9:**
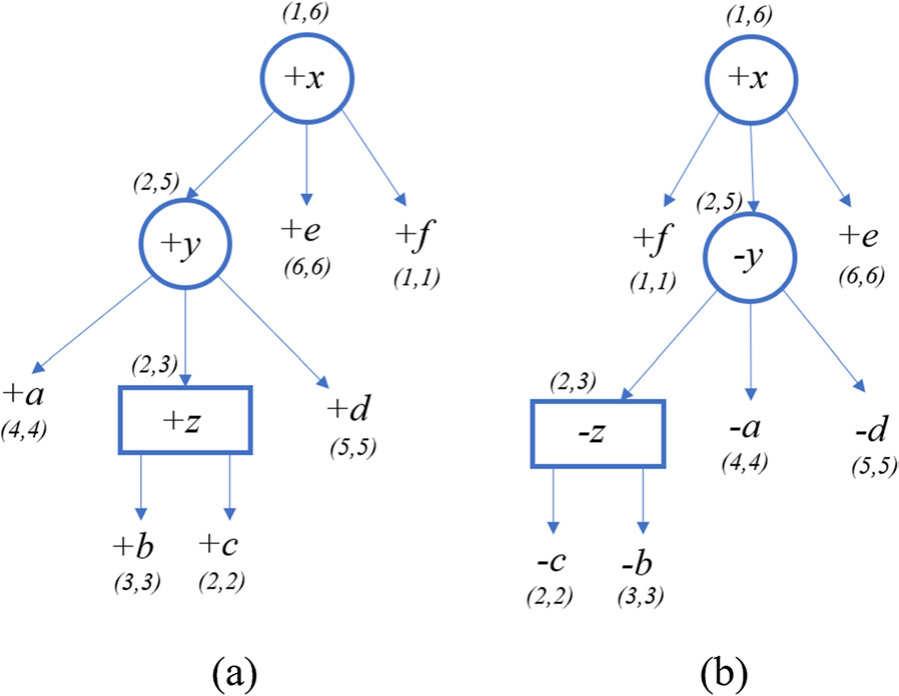
**a** PQ-tree *T*. **b** PQ-tree $T'$, which is quasi-equivalent to *T* and ordered as $S_2$

Finally, consider the iteration where $\mathcal {A}[x,1]$ is calculated (the values for all other entries with node *x* is $\infty$). Figure 8 describes *T* and $T'$ after calculating the entries of the children of the P-node *x*. The intervals of the children complete the interval (1, *e*), where $e=1+\textsf{span}(x)-1=6$, so we can continue with the iteration. $childrenDist=\mathcal {A}[y,2]+\mathcal {A}[e,6]+\mathcal {A}[f,1]=10$. $\mathsf {d_{SBP}}(S(x), S(x'))=2$ where the pairs (*y*, *e*) and (*e*, *f*) are the signed break-points (note that $S(x)=+y+e+f$, $S(x')=+f-y+e$). $\Delta ^P_{\textsf{jump}}(x,x')=0$ because to reorder the children of *x* as $S_2$ we can move *f* only (and its size is 1). Therefore, $\Delta ^{\textsf{P}}_{\textsf{violation}}(x,x') = \mathsf {d_{SBP}}(S(x), S(x')) + \Delta ^P_{\textsf{jump}}(x,x')=2$. Then, $\mathcal {A}[x,1]=10+2=12$ and the algorithm returns 12. Figure 9 describes the PQ-tree *T* and its quasi-equivalent PQ-tree $T'$ after calculating $\mathcal {A}[x,1]$.

### Complexity analysis

In this section we analyse the time and space complexities of Algorithm 1, which solves $\textsf{CTTSD}{}$. First, we analyse Algorithms 2 and 3, which are used as procedures in Algorithm 1. For a given PQ-tree, we denote by $\gamma$ the maximum number of children of an internal node.

#### **Lemma 5.1**

*Algorithm 5 takes* $O(1.381^\gamma \gamma ^2)$* time and *$O(\gamma ^2)$* space.*

#### *Proof*

The most space consuming part of the algorithm, besides the computation of a vertex cover, is to store $x'$ (which is equivalent to *x* and ordered as a specific string). We simply save the children of *x* in a specific order. Thus, the space is $O(\gamma )$.

The algorithm first checks if the interval $[\ell ,e]$ can be “completed” by all of the intervals defined by the indices of the children of *x*. We can check this in $O(\gamma ^2)$ time (naively). Then, we sum up the values in $\mathcal {D}{}$. Notice that $|\mathcal {D}{}|=|\textsf{children}(x)|=O(\gamma )$, thus this step takes $O(\gamma )$ time.

After that, we calculate $\Delta ^{\textsf{P}}_{\textsf{violation}}(x,x')$, which its most time consuming calculation is the *jump violation*, $\Delta ^P_{\textsf{jump}}(x,x')$, which requires to find a minimum weighted vertex cover of the graph ${G[x,x']}$. In order to find such vertex cover, we can use, for example, the algorithm in [44] which takes $O(1.381^\gamma \gamma ^2)$ time and $O(\gamma ^2)$ space. Thus, this step takes $O(1.381^\gamma \gamma ^2)$ time and $O(\gamma ^2)$ space.

All other steps in the algorithm are basic operations and thus they take *O*(1) time. Hence, the algorithm takes $O(1.381^\gamma \gamma ^2)$ time and $O(\gamma ^2)$. $\square$

#### **Lemma 5.2**

*The Q-Mapping algorithm, Algorithm 6, takes* $O(\gamma ^2)$ *time and* $O(\gamma )$ *space.*

#### *Proof*

The most space consuming part of the algorithm is to store $x'$ (which is equivalent to *x* and ordered as a specific string). We simply save the children of *x* in a specific order. Thus, the space of the algorithm is $O(\gamma )$.

The algorithm first checks if the interval $[\ell ,e]$ can be “completed consecutively” by all of the intervals defined by the indices of the children of *x*. We can check this in $O(\gamma )$ time. Then, we sum up the values in $\mathcal {D}{}$. Notice that $|\mathcal {D}{}|=|\textsf{children}(x)|=O(\gamma )$, thus this step takes $O(\gamma )$ time. After that, we calculate $\Delta ^{\textsf{Q}}_{\textsf{violation}}(x,x')$ and $\textsf{FlipCorrection}(x,x')$, which take $O(\gamma ^2)$ time (naively). All other steps in the algorithm are basic operations and thus they take *O*(1) time. Hence, the algorithm takes $O(\gamma ^2)$ time. $\square$

#### **Lemma 5.3**

*The main algorithm, Algorithm 1, takes* $O(n \gamma ^2 \cdot (m_p \cdot 1.381^\gamma + m_q))$* time and *$O(n^2)$* space.*

#### *Proof*

The number of leaves in the PQ-tree *T* is *n*, hence there are *O*(*n*) nodes in the tree, thus the size of the first dimension of the DP table, $\mathcal {A}{}$, is *O*(*n*). The size of the second dimension ($1 \le \ell \le n$) is also *n*. Thus, the DP table $\mathcal {A}{}$ is of size $O(n^2)$.

In the initialization step, all calculations are basic and take *O*(1) time. The P-Mapping algorithm is called for every P-node in *T* and every possible start index *i*, so the P-Mapping algorithm is called $O(n m_p)$ times. Similarly, the Q-Mapping algorithm is called $O(n m_q)$ times. Thus, it takes $O(n \cdot (m_p \cdot \text {Time(P-Mapping)} + m_q \cdot \text {Time(Q-Mapping)}))$ time to fill the DP table. In the final stage of the algorithm, we return $\mathcal {A}[root_T,1]$, which takes *O*(1) time.

From Lemma 5.1, the P-Mapping algorithm takes $O(1.381^\gamma \gamma ^2)$ time and $O(\gamma ^2)$ space, and from Lemma 5.2, the Q-Mapping algorithm takes $O(\gamma ^2)$ time and $O(\gamma )$ space. Thus, in total, our algorithm runs in $O(1) + O(n \cdot (m_p \cdot (2^\gamma \gamma ^2) + m_q \cdot (\gamma ^2))) = O(n \gamma ^2 \cdot (m_p \cdot 1.381^\gamma + m_q))$ time. Adding the space required for the P-Mapping and Q-Mapping algorithms to the space required for the main DP table results in a total space complexity of $O(\gamma ^2)+O(\gamma )+O(n^2) = O(n^2)$. $\square$

Observe that is $\Delta ^P_{\textsf{jump}}$ is set to 0, then the computation of a vertex cover is not required. Hence, by the analysis above, we obtain the following time and space complexities.

#### **Lemma 5.4**

*If *$\Delta ^P_{\textsf{jump}}$* is set to 0, then the main algorithm, Algorithm 1, takes* $O(n m \gamma ^2)$* time and* $O(n^2)$* space.*

## $\mathsf {TreeToString \ Divergence}$: algorithm

In this section, we develop a dynamic programming (DP) algorithm to solve $\textsf{TTSD}{}$. Our algorithm receives as input an instance of $\textsf{TTSD}{}$, $(S_1,S_2,T,d_T,d_S,\delta ^Q_{\textsf{ord}},\delta ^Q_{\textsf{flip}})$. The output of the algorithm is the minimum divergence from *T* to a subsequence of $S_2$ with up to $d_T$ deletions from *T* and up to $d_S$ deletions from the subsequence (or “NO”).

Brief overview: On a high level, our algorithm consists of three components: the main algorithm, and two other algorithms that are used as procedures by the main algorithm. Apart from an initialization phase, the crux of the main algorithm is a loop that traverses the given PQ-tree, *T*. For each internal node *x*, it calls one of the two other algorithms: P-Mapping or Q-Mapping. These algorithms return the divergence from the subtree of *T* rooted in *x*, $T_x$, to subsequences of *S*, based on the type of *x* (P-node or Q-node). Then, these divergences are stored in the DP table.

In the following sections, we describe the main algorithm (“The main algorithm” section), the P-Mapping algorithm (“P-node mapping: the algorithm” section) and the Q-Mapping algorithm (“Q-node mapping: the algorithm” section).

### The main algorithm

The algorithm (whose pseudocode is given in Algorithm 4) constructs a 5-dimensional DP table $\mathcal {A}{}$ of size $m' \times m \times n \times d_T+1 \times d_S+1$, where $m'=m+m_p+m_q$ is the number of nodes in *T*. The purpose of an entry of the DP table, $\mathcal {A}[x,pos,i,k_T,k_S]$, is to hold the divergence from the subtree $T_x$ to a subsequence $S'$ of $S_2$ starting at index *i* with $k_T$ deletions from $T_x$ and $k_S$ deletions from $S'$, where exactly *pos* leaves of $T_x$ have a positive sign. If $S'$ cannot be derived from $T_x$, $\mathcal {A}[x,pos,i,k_T,k_S] = \infty$.

Some entries of the DP table define illegal derivations, namely, derivations for which the number of deletions is inconsistent with the start index *i*, the derived node and $S_2$. For example, such are derivations that have more deletions from the string than there are characters in the derived string. These entries are called *invalid entries*, and their value is defined as $\infty$ throughout the algorithm. Formally, an entry $\mathcal {A}[x,pos,i,k_T,k_S]$ is invalid if one of the following is true: $pos > \textsf{span}(x)-k_T$, $k_T > \textsf{span}(x)$, $k_S > L(x,k_T,k_S)$, or $E(x,i,k_S,k_T) > n$.

Let *x* be a leaf, *S* be a signed string, *i* be an index, $k_S$ be the number of deletions from *S* and $pos \in \{0,1\}$. Then, we define $I_{x,S,i,k_S,pos}=\{i \le j \le i+k_S \, \ \textsf{label}(x)=S[j]$ and *pos* is consistent with $\textsf{sign}(S[j]) \}$. Intuitively, each $j \in I_{x,S,i,k_S,pos}$ corresponds to a possible alignment of *x* to *S*[*j*], therefore the label of *x* must match *S*[*j*] and *pos* must be consistent with $\textsf{sign}(S[j])$.

The algorithm first initializes the entries of $\mathcal {A}{}$ that are meant to hold scores of derivations of the leaves of *T* to every possible subsequence of *S* using the following rule. For every $0\le k_S\le d_S$, every leaf $x \in \textsf{Leaves}(T)$ and each possible sign of *y* ($pos \in \{0,1\}$), do:

1. $$\mathcal {A}[x,pos,i,1,k_S] = \rho ^T_{\textsf{del}}+ k_S \cdot \rho ^S_{\textsf{del}}$$ (if $$d_T>0$$).
2. $\mathcal {A}[x,pos,i,0,k_S] = \infty$ if there is no derivation from *x* to a character in $S_2[i:i+k_T]$.
  Otherwise:
  - $\mathcal {A}[x,pos,i,0,k_S] = 0$ if *pos* is consistent with $\textsf{sign}(x)$ (no flipping).
  - $\mathcal {A}[x,pos,i,0,k_S] = \delta ^Q_{\textsf{flip}}$ if *pos* is not consistent with $\textsf{sign}(x)$ (flipping).

After the initialization, all other entries of $\mathcal {A}{}$ are filled as follows. Go over the internal nodes of *T* in postorder. For every internal node *x*, go in ascending order over every index *i* that can be a start index for a subsequence of $S_2$ derived from $T_x$ (the possible values of *i* are explained in the next paragraph). For every *x* and *i*, use the algorithm for P-mapping or Q-mapping according to the type of *x*. Both algorithms receive the following input: a subsequence $S'$ of $S_2$, the node *x*, its children $x_{1},\dots ,x_{\gamma }$, the collection of all possible derivations of the children (denoted by $\mathcal {D}{}$), which have already been computed and stored in $\mathcal {A}{}$ (as will be explained ahead), and the deletion arguments $d_T,d_S$. Q-Mapping also receives the penalty arguments $\delta ^Q_{\textsf{ord}}$ and $\delta ^Q_{\textsf{flip}}$ as input. Intuitively, the subsequence $S'$ is the longest subsequence of *S* starting at index *i* that can be derived from $T_x$ given $d_T$ and $d_S$. After being called, both algorithms return a set of divergences of derivations of $T_x$ to a prefix of $S'=S[i:e]$. The set holds the divergences of derivations for every $E(x,i,d_T,0) \le e \le E(x,i,0,d_S)$ and for every $0\le k_T\le d_T$, $0\le k_S \le d_S$.
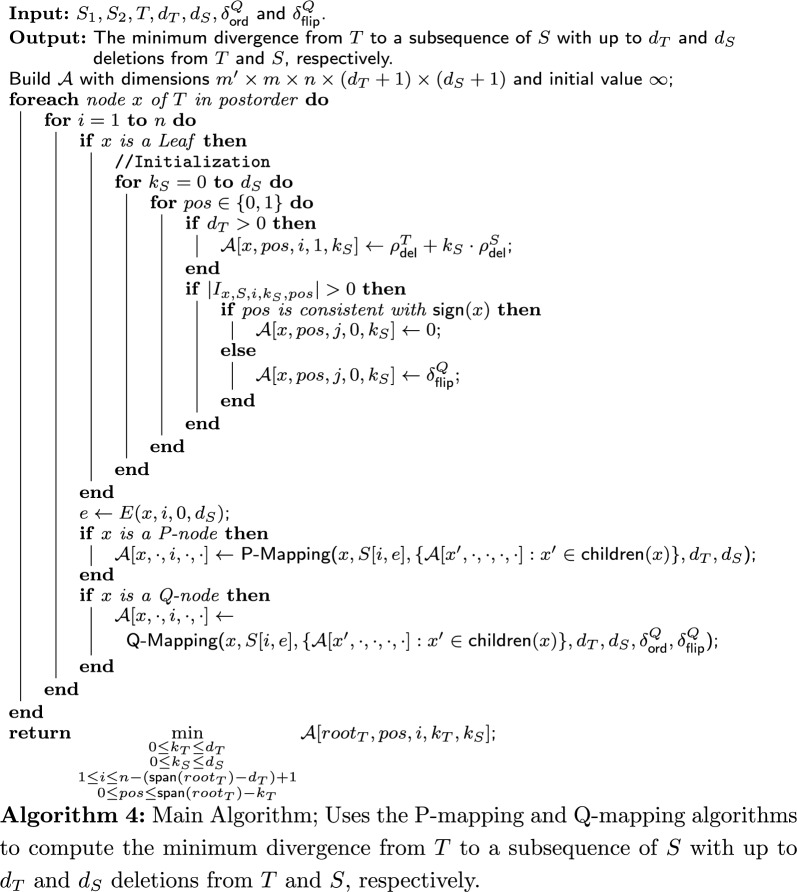


We now explain the possible values of *i* and the definition of $S'$ more formally. To this end, recall the length function given in Definition 18, $L(x,k_T,k_S) = \textsf{span}(x) - k_T + k_S$. Thus, on the one hand, a subsequence of maximum length is obtained when there are no deletions from the tree and $d_S$ deletions from the string. Hence, $S'=S[i:E(x,i,0,d_S)]$. On the other hand, a shortest subsequence is obtained when there are $d_T$ deletions from the tree and none from the string. Then, the length of the subsequence is $L(x,d_T,0) = \textsf{span}(x)-d_T$. Hence, the index *i* runs between 1 and $n-(\textsf{span}(x)-d_T)+1$.

We now turn to address the aforementioned input collection $\mathcal {D}{}$ in more detail. Formally, it contains derivations of every child $x'$ of *x* to every subsequence of $S'$ with up to $d_T$ and $d_S$ deletions from the tree and string, respectively. It is obtained from the entries $\mathcal {A}[x',pos', i', k_T, k_S]$ (where each entry yields one derivation) for all $k_T$ and $k_S$, all $i'$ between *i* and the end index of $S'$, i.e., $i\le i' \le E(x,i,0,d_S)$, and all possible $pos'$ ($0 \le pos' \le \textsf{span}(x)-k_T$).

In the final stage of the main algorithm, when the DP table is full, the score of a best derivation is the minimum of $\{\mathcal {A}[root_T,pos,i,k_T,k_S]: k_T\le d_T, k_S \le d_S, 1\le i\le n-(\textsf{span}(root_T)-k_T)+1, 0 \le pos \le \textsf{span}(root_T)-k_T\}$.

### P-node and Q-node mapping: terminology

Before describing the P-mapping and Q-mapping algorithms, we set up some useful terminology.

We first define the notion of a *partial derivation*. In the P-Mapping and Q-Mapping algorithms, derivations of the input node *x* are built by considering subsets *C* of its children. With respect to such a subset *C*, a derivation $\mu$ of *x* is built as if *x* had only the nodes in *C* as children, and is called a *partial derivation*. We denote by $\mu .v$ the root of the subtree of the derivation ($T_{\mu .v}$). $\mu .pos$ denotes the number of positive signed of leaves considered in the derivation (in $T_{\mu .v}$). $\mu .del_T$ and $\mu .del_S$ denote the number of deletions from $T_{\mu .v}$ and from the string in the derivation, respectively. $\mu .score$ denotes the divergence score of the derivation $\mu$.

#### **Definition 20**

Let *x* be a node. $\mu$ is a partial derivation between *x* and a string if $\mu .v=x$ and there is a subset of children $C \subseteq \textsf{children}(x)$ such that the two following conditions are true.

1. For every $u \in \textsf{children}(x) \setminus C$, each of the leaves in $T_u$ is neither mapped nor deleted under $\mu$.
2. For every $u \in C$, each of the leaves in $T_u$ is either mapped or deleted under $\mu$.

For every $u \in \textsf{children}(x) \setminus C$, we say that *u* is *ignored under* $\mu$. Notice that any derivation is a partial derivation, where the set of ignored nodes ($U'$ above) is empty.

In the P-Mapping algorithm for $C \subseteq \textsf{children}(x)$, the notation $x^{(C )}$ is used to indicate that the node *x* is considered as if its only children are the nodes in *C* (the nodes in $\textsf{children}(x)\setminus C$ are ignored). Consequentially, the span of $x^{(C )}$ is defined as $\textsf{span}(x^{(C )}) = \sum _{c\in C}\textsf{span}(c)$, and the set $\mathcal {D}(C,N,y,k_T,k_S){}$ (in Definition 21 where $U=\{x^{(C )}\}$) now refers to a set of *partial* derivations. To use $x^{(C )}$ to describe the base cases of the algorithm, let us define $x^{(\emptyset )}$ ($x^{(C )}$ for $C=\emptyset$) as a tree with no labeled leaves to map.

Since all derivations that are computed in a single call to the P-Mapping algorithm have the same start-point *i*, it can be omitted (for brevity) from the end-point function; thus, we denote $E(x,k_T,k_S) = L(x,k_T,k_S)$. Also, for a set *U* of nodes, we define $L(U,k_T,k_S) = \sum _{x\in U}\textsf{span}(x)+k_S-k_T$, and, accordingly, $E(U,k_T,k_S) = L(U,k_T,k_S)$.

We now define certain collections of derivations with common properties (such as having the same number of deletions and end-point).

#### **Definition 21**

Let *C* be a set of nodes, $N \subseteq C$ be the set of nodes which have negative sign among the nodes in *C*, and $k_T$ and $k_S$ be two numbers. The collection of all the derivations of $y \in C$ to suffixes of $S'[1:E(C,k_T,k_S)]$ with *exactly* $k_T$ deletions from the tree and *exactly* $k_S$ deletions from the string and are consistent with *N* is denoted by $\mathcal {D}(C,N,y,k_T,k_S){}$. By consistency with *N* we mean: if $y \in N$, then for each $\mu \in \mathcal {D}(C,N,y,k_T,k_S){}$, $\mu .pos \le (\textsf{span}(y)-k_T)/2$; if $y \notin N$, then for each $\mu \in \mathcal {D}(C,N,y,k_T,k_S){}$, $\mu .pos \ge (\textsf{span}(y)-k_T)/2$.

#### **Definition 22**

Let *C* be a set of nodes, $N \subseteq C$ be a set of nodes which define the signs of the nodes in *C*, and $k_T$ and $k_S$ be two numbers. The collection of all derivations of $y \in C$ to suffixes of $S'[1:E(C,k_T,k_S)]$ with *up to* $k_T$ deletions from the tree, and *up to* $k_S$ deletions from the string is denoted by $\mathcal {D}_\le {(C,N,y,k_T,k_S)}$. Specifically, for the node $y \in C$, $k'_T\le k_T$ and $k'_S\le k_S$, the set $\mathcal {D}_\le {(C,N,y,k_T,k_S)}$ holds only one derivation of *y* to a suffix of $S'[1:E_{I}(C,k_T,k_S)]$ with $k'_T$ and $k'_S$ deletions from the tree and string, respectively, if such derivation exists. In addition, the derivations in $\mathcal {D}_\le {(C,N,k_T,k_S)}$ are consistent with *N*: if $y \in N$, then for each $\mu \in \mathcal {D}_\le {(C,N,y,k_T,k_S)}$, $\mu .pos \le (\textsf{span}(y)-k_T)/2$; if $y \notin N$, then for each $\mu \in \mathcal {D}_\le {(C,N,y,k_T,k_S)}$, $\mu .pos \ge (\textsf{span}(y)-k_T)/2$.

It is important to distinguish between these two definitions. First, the derivations in $\mathcal {D}(C,N,y,k_T,k_S){}$ have *exactly* $k_T$ and $k_S$ deletions, while the derivations in $\mathcal {D}_\le {(C,N,y,k_T,k_S)}$ have *up to* $k_T$ and $k_S$ deletions. Second, in $\mathcal {D}(C,N,y,k_T,k_S){}$ there can be several derivations that differ only in their scores and in the one-to-one mappings that yield them, while in $\mathcal {D}_\le {(C,N,y,k_T,k_S)}$ there is only one derivation for every deletion combination pair $(k'_T,k'_S)$. Note that the end-points of all of the derivations are equal.

In every step of the P-Mapping algorithm, a different set of derivations of the children of *x* is examined, thus, Definition 22 is used for $C \subseteq \textsf{children}(x)$. In addition, the set of derivations $\mathcal {D}{}$ that is received as input to the algorithms can be described using Definition 22 as can be seen in Eq. 1 below. In this equation, the union is over all $C\subseteq \textsf{children}(x)$ because in this way the derivations of all the children of *x* with *every possible end-point* are obtained (in contrast to having only $C=\textsf{children}(x)$, which results in the derivations of all the children of *x* with the end-point $E(\textsf{children}(x),k_T,k_S)$).

1 $$\begin{aligned} \mathcal {D}{} = \bigcup _{C \subseteq \textsf{children}(x)}\bigcup _{N \subseteq C}\bigcup _{y \in C}\bigcup _{k_T\le d_T}\bigcup _{k_S\le d_S} \mathcal {D}_\le {(C,N,y,k_T,k_S)} \end{aligned}$$

In the P-Mapping algorithm for $C \subseteq \textsf{children}(x)$, the notation $x^{(C )}$ is used to indicate that the node *x* is considered as if its only children are the nodes in *C* (the nodes in $\textsf{children}(x)\setminus C$ are ignored). Consequentially, the span of $x^{(C )}$ is defined as $\textsf{span}(x^{(C )}) = \sum _{c\in C}\textsf{span}(c)$, and the set $\mathcal {D}(C,N,y,k_T,k_S){}$ (in Definition 21 where $C=\{x^{(C )}\}$) now refers to a set of *partial* derivations. To use $x^{(C )}$ to describe the base cases of the algorithm, let us define $x^{(\emptyset )}$ ($x^{(C )}$ for $C=\emptyset$) as a tree with no labeled leaves to map.

In the P-Mapping algorithm, we use the following notation.

#### **Definition 23**

Let *C* be a set of children of a P-node in a PQ-tree. For $x,y \in C$, the set of all nodes in *C* that are between *x* and *y* is denoted by $C_{x,y}$. That is, $C_{x,y} = \{v \in C: v \ is \ between \ x \ and \ y\}$.

**Fig. 10:**
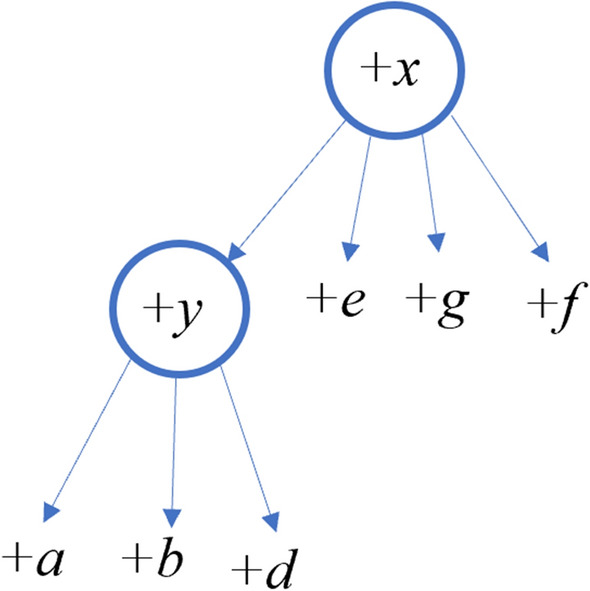
$$C_{y,f}=\{e,g\}, C_{a,d}=\{b\}, C_{y,e}=\{\}$$

See an example in Fig. 10.

In the algorithms, we take in account signed break-points between the nodes in the derivations. So, we define a procedure which receives a node *x*, two children of *x*, *y* and *z* that are considered to be adjacent in a quasi-equivalent tree $T_{x'}$ of $T_x$ and a set *N* that defines the signs of *y* and *z* in $T_{x'}$. The procedure returns 1 if there is a signed break-point between *y* and *z*, and 0 otherwise. The procedure checks if the nodes are adjacent in the tree $T_x$ and if they changed their signed order (recall Definition 9) according to *N*, as defined next.

#### **Definition 24**

2 $$\begin{aligned} \textsf{BPDelta}(x,y,z,N) = {\left\{ \begin{array}{ll} 0, &{} if \ y \ and \ z \ are \ adjacent \ in \ T_x \ and \ did \ not \ change \ their \ signed \ order \\ 1, &{} else \end{array}\right. } \end{aligned}$$

Similar to Definition 24, we define a procedure which is used in a case where we necessarily consider the two children *y*, *z* of *x* to be adjacent in the tree $T_x$ (specifically, when $C_{y,z}$ is deleted). This procedure returns 1 if there is a signed break-point between *y* and *z*, but does not check if they are adjacent.

#### **Definition 25**

3 $$\begin{aligned} \textsf{BPDelta2}(x,y,z,N) = {\left\{ \begin{array}{ll} 0, &{} if \ y \ and \ z \ did \ not \ change \ their \ signed \ order \\ 1, &{} else \end{array}\right. } \end{aligned}$$

In the P-Mapping algorithm, we calculate the *jump violation* (recall Definition 12). To do so, we define the procedure $\textsf{JumpViolationDelta}$, which receives a vertex cover *C* and a node *x*, and returns the penalty defined in Definition 12 of *x* as follows.

#### **Definition 26**

4 $$\begin{aligned} \textsf{JumpViolationDelta}(C,x) = {\left\{ \begin{array}{ll} (\textsf{span}(x)-1)/2, &{} if \ x \in C \\ 0, &{} else \end{array}\right. } \end{aligned}$$

### P-node mapping: the algorithm

Recall that the input consists of a P-node *x*, a string $S'$, bounds on the number of deletions from *T* and $S'$, $d_T$ and $d_S$, respectively, and a set of derivations $\mathcal {D}{}$ (see Eq. 1). The output of the algorithm is the collection of divergences of derivations of *x* to every prefix of $S'$ having exactly $k_T$ deletions from $T_x$, $k_S$ deletions from the prefix of $S'$ and *pos* number of leaves with positive sign, for each combination of $0 \le k_T \le d_T$, $0 \le k_S \le d_S$ and $0 \le pos \le \textsf{span}(x)$. Thus, the output contains $O(d_T \cdot d_S \cdot \textsf{span}(x))$ derivations.

The algorithm (whose pseudocode is given in Algorithm 5) constructs a 7-dimensional DP table $\mathcal {P}{}$, which has an entry for every $0\le k_T \le d_T$, $0\le k_S \le d_S$, subset $C\subseteq \textsf{children}(x)$, subset $C'\subseteq C$, subset $N\subseteq C$, number $0 \le pos \le \textsf{span}(x)$ and for every $y \in C$. Recall that we need to calculate $\textsf{JumpViolation}$ for the children of *x*. To do so, we use the variables $C'$ and *N*. The purpose of an entry $\mathcal {P}[C,C',N,pos,y,k_T,k_S]$ is to hold the divergence of a partial derivation rooted in $x^{(C )}$ to a prefix of $S'$ with exactly $k_T$ deletions from the tree, $k_S$ deletions from the string, *pos* leaves with positive sign in $T_{x'^{(C)}}$, where $x'$ is the node equivalent to *x*, while considering the nodes in *N* to have a negative sign, $C'$ as a possible vertex cover set of $G[x^{(C )},x'^{(C)}]$ (minus the children that were deleted), and considering derivations of *y* only to suffixes of $S'[1:E(C,k_T,k_S)]$. The children of *x* that are not in *C* are *ignored* under the partial derivation stored by the DP table entry $\mathcal {P}[C,C',N,pos,y,k_T,k_S]$, thus they are neither deleted nor counted in the number of deletions from the tree, $k_T$. (They will be accounted for in the computation of other entries of $\mathcal {P}{}$).

Similarly to the main algorithm, some of the entries of $\mathcal {P}{}$ are invalid, and their value is defined as $\infty$. Formally, an entry $\mathcal {P}[C,C',N,pos,y,k_T,k_S]$ is invalid if one of the following is true: $k_T > \textsf{span}(C)$, $L(x^{(C )},k_T,k_S) > \textsf{len}(S')$, *pos* is not consistent with *N*, or $C'$ is not a vertex cover of $G[x^{(C )},x'^{(C)}]$ where the signs of $\textsf{children}(x'^{(C)})$ are defined by *N*.

Every entry $\mathcal {P}[C,C',N,pos,y,k_T,k_S]$ for which $|C|=1$ is initialized with $\mathcal {D}_{(C,N,y,pos,k_T,k_S)}$ (stored in $\mathcal {D}{}$) which is the divergence of the derivation rooted in $T_y$ to the suffix of $S'[1:E(C,k_T,k_S)]$, with exactly $k_T$ deletions from the tree, $k_S$ deletions from the string, *pos* leaves with positive sign in $T_{y'}$ such that *N* is consistent with *pos* (if it exists, this derivation is stored in $\mathcal {D}{}$). If such a derivation does not exist, $\mathcal {D}_{(C,N,y,pos,k_T,k_S)} = \infty$.

After the initialization, the remaining entries of $\mathcal {P}{}$ are calculated using the recursion rule in Expression 1 ahead. The order of computation is ascending with respect to the size of the subsets *C* of the children of *x*, and for a given $C\subseteq \textsf{children}(x)$, the order is ascending with respect to the number of deletions from both tree and string. With a lesser priority, the order is ascending with respect to the number of positive signed leaves in $T_{x'^{(C)}}$.
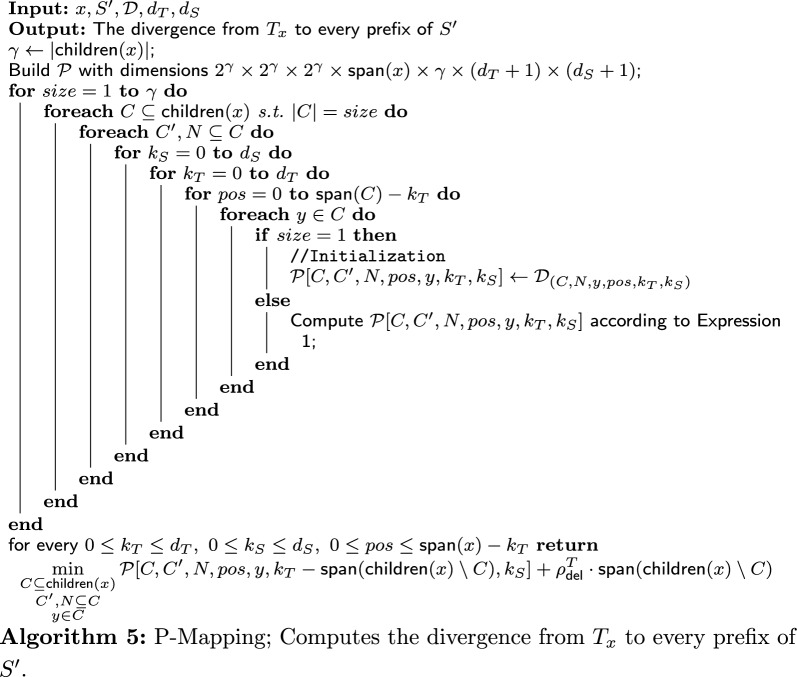


In the algorithm, $\mathcal {P}[C,C',N,pos,y,k_T,k_S]$ is computed by taking the minimum between the following expressions (we will refer the minimum of the three expressions as Expression 1):

1. $\mathcal {P}[C,C',N,y,k_T,k_S-1] + \rho ^S_{\textsf{del}}$ Explanation: Intuitively, every entry $\mathcal {P}[C,C',N,pos,y,k_T,k_S]$ defines some index $e'$ of $S'$ that is the end-point of every partial derivation in $\mathcal {D}(C,N,y,k_T,k_S){}$. Thus, $S'[e']$ must be a part of any partial derivation $\mu \in \mathcal {D}(C,N,y,k_T,k_S){}$; so, either $S'[e']$ is deleted under $\mu$ or it is mapped under $\mu$. The former option is captured by the first case of the recursion rule.
2. $$\displaystyle \min _{\begin{array}{c} \mu \in \mathcal {D}_\le {(C,N,y,k_T,k_S)} \\ s.t. \ \mu .del_T<\textsf{span}(y) \end{array}} \displaystyle \min _{z \in {C{\setminus } \{y\}}} \mathcal {P}[C{\setminus } \{y\},C'{\setminus } \{y\},N{\setminus } \{y\},pos-\mu .pos,z,k_T-\mu .del_T, k_S-\mu .del_S] + \mu .score + \textsf{BPDelta}(x,y,z,N) + \textsf{JumpViolationDelta}(C',y)$$
  Explanation: If $S'[e']$ is mapped under $\mu$, then due to the hierarchical structure of $T_x$, it must be mapped under some derivation $\mu '$ of one of the children of *x* that are in *C*. Thus, we receive the second and third cases of the recursion rule. In the second case, we consider $z \in C\setminus \{y\}$ and derivations of *z* only to suffixes of $S'[1:E(C\setminus \{y\},k_T-\mu .del_T,k_S-\mu .del_S)]$.
3. $$\displaystyle \min _{\begin{array}{c} \mu \in \mathcal {D}_\le {(C,N,y,k_T,k_S)}\\ s.t. \ \mu .del_T<\textsf{span}(y) \end{array}} \displaystyle \min _{\begin{array}{c} z \in {C{\setminus } \{y\}} \\ s.t. C_{y,z}\subseteq C \end{array}} \mathcal {P}[C{\setminus } C_{y,z} {\setminus } \{y\},C'{\setminus } C_{y,z} {\setminus } \{y\},N{\setminus } C_{y,z} {\setminus } \{y\},z,k_T-\mu .del_T- \textsf{span}(C_{y,z}), k_S-\mu .del_S] + \mu .score + \rho ^T_{\textsf{del}}\cdot \textsf{span}(C_{y,z}) + \textsf{BPDelta2}(x,y,z,N) + \textsf{JumpViolationDelta}(C',y)$$
  Explanation: The third case captures the option of deletion of all the nodes between *y* and *z* ($C_{y,z}$), so that after the deletion we consider *y* and *z* as adjacent in $T_{x^{(C )}}$. In this case we consider $z \in C\setminus \{y\}$ and derivations of *z* only to suffixes of $S'[1:E(C{\setminus } C_{y,z} {\setminus } \{y\},k_T-\mu .del_T- \textsf{span}(C_{y,z}),k_S-\mu .del_S)]$ (1)

Once the entire DP table is filled, for every combination of $(pos,k_T,k_S)$ the algorithm returns the divergence from $T_x$ to $S'$. This is done by going over the partial derivations in $\mathcal {P}{}$ as well as deleting the ignored nodes (and penalizing the deletion accordingly).

### Q-node mapping: the algorithm

The input and output of the Q-Mapping algorithm are the same as the input and output of the P-mapping algorithm (“P-node mapping: the algorithm” section), respectively, except for the type of the node *x* received as input, and the penalty parameters $\delta ^Q_{\textsf{ord}}$ and $\delta ^Q_{\textsf{flip}}$, which are also part of the input.

Given that the children of *x* in consecutive order are $x_1,x_2,\dots ,x_\gamma$ and given an index $1\le i\le \gamma$, $x_{[i]}$ denotes the set of the first *i* children of *x*. Formally, $x_{[i]} = \{x_1,\dots ,x_i\}$. In addition, $x^{(i)}$, denotes the node *x* as if its only children are the nodes in $x_{[i]}$. Consequentially, the span of $x^{(i)}$ is defined as $\sum _{j=1}^i{\textsf{span}(x_j)}$ and the set $\mathcal {D}(x^{(i)},N,y,k_T,k_S){}$ (see Definition 21 where $C=\{x_{[i]}\}$) now refers to a set of *partial* derivations. In addition, $x_{[i:j]}$ denotes the set of children of *x* from child *i* to child *j*; formally, $x_{[i:j]} \doteq \{x_i,\dots ,x_j\}$. In case $i>j$, $x_{[i:j]}=\emptyset$.

The algorithm (whose pseudocode is given in Algorithm 6) constructs two 5-dimensional DP tables $\mathcal {Q}_\ell$ and $\mathcal {Q}_r$. Both have an entry for every $0\le k_T \le d_T$, $0\le k_S \le d_S$, index $0\le i \le \gamma$, $s \in \{+,-\}$ and number $0 \le pos \le \textsf{span}(x)$. The purpose of an entry $\mathcal {Q}_\ell [i,k_T,k_S,s,pos]$ (and similarly $\mathcal {Q}_r[i,k_T,k_S,s,pos]$) is to hold the divergence of a partial derivation in $\mathcal {D}(x^{(i)},N,y,k_T,k_S){}$, i.e. a partial derivation rooted in $x^{(i)}$ to a prefix of $S'$ with exactly $k_T$ deletions from the tree, $k_S$ deletions from the string, while *s* is the sign of $x_i$ in the derivation and *pos* is the number of positive signed leaves in $x^{(i)}$. The difference between $\mathcal {Q}_\ell$ and $\mathcal {Q}_r$ is in the order in which the children of *x* are arranged. In $\mathcal {Q}_\ell$ the children of *x* are considered in a left-to-right order, namely, $x_1$ is the leftmost child of *x* and $x^\ell _{[i]}$ is the set of the *i* leftmost children of *x* (then, $x^\ell _{[i:j]}$ is defined accordingly). In $\mathcal {Q}_r$ the children of *x* are considered in a right-to-left order, namely, $x_1$ is the rightmost child of *x* and $x^r_{[i]}$ is the set of the *i* rightmost children of *x* (then, $x^r_{[i:j]}$ is defined accordingly). For abbreviation, from now on, $\mathcal {Q}{}$ is used when a notion is true for both $\mathcal {Q}_\ell$ and $\mathcal {Q}_r$. The children of *x* that are not in $x_{[i]}$ are *ignored* under the partial derivation stored by the DP table entry $\mathcal {Q}[i,k_T,k_S,s,pos]$, thus they are neither deleted nor counted in the number of deletions from the tree, $k_T$. (They will be accounted for in the computation of other entries of $\mathcal {Q}{}$).

Similarly to the main algorithm and the P-Mapping algorithm, some of the entries of the DP tables are invalid, and their value is defined as $\infty$. Formally, an entry $\mathcal {Q}[i,k_T,k_S,s,pos]$ is invalid if one of the following is true: $k_T > \textsf{span}(x^{(i)})$, $L(x_{[i]},k_T,k_S) > |S'|$, or *pos* is not consistent with *s*.
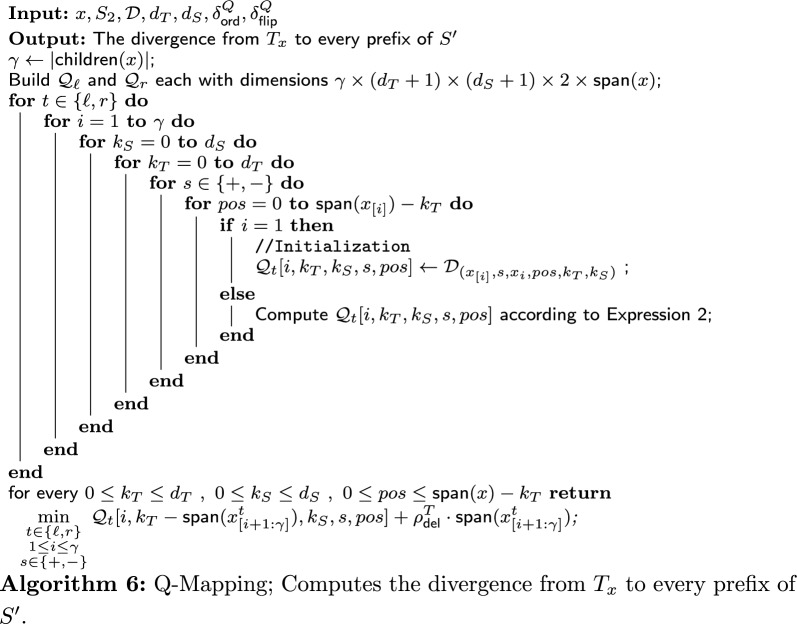


Every entry $\mathcal {Q}[i,k_T,k_S,s,pos]$ for which $i=1$ is initialized with $\mathcal {D}_{(x_{[i]},s,x_i,pos,k_T,k_S)}$ (stored in $\mathcal {D}{}$) which is the divergence of the derivation rooted in $T_{x_i}$ to the suffix of $S'[1:E(x_{[i]},k_T,k_S)]$, with exactly $k_T$ deletions from the tree, $k_S$ deletions from the string, *pos* leaves with positive sign in $T_{x'_i}$ such that *s* is consistent with *pos* (if it exists, this derivation is stored in $\mathcal {D}{}$). If such a derivation does not exist, $\mathcal {D}_{(x_{[i]},s,x_i,pos,k_T,k_S)} = \infty$.

After the initialization, the remaining entries of $\mathcal {Q}{}$ are calculated using the recursion rule in Expression 2 ahead. The order of computation is ascending with respect to the child index *i*, and for a given *i*, the order of computation is ascending with respect to the number of deletions from the string, and ascending with respect to the number of positive signed leaves in $T_{x'^{(i)}}$. In Expression 2 we use the notation $N_{x_i}$ and $N_{x_i,x_j}$, defined as follows. Let $x_i$ and $x_j$ be two nodes whose signs in $T_{x'^{(i)}}$ are *s* and $s'$, respectively. If $s=-$ then $x_i \in N_{x_i}$ and $x_i \in N_{x_i,x_j}$, and if $s'=-$ then $x_j \in N_{x_i,x_j}$. If $s=+$ then $x_i \notin N_{x_i}$ and $x_i \notin N_{x_i,x_j}$, and if $s'=+$ then $x_j \notin N_{x_i,x_j}$.

In the algorithm, $\mathcal {Q}_t[i,k_T,k_S,s,pos]$ is computed by taking the minimum between the following expressions (we will refer the minimum of the three expressions as Expression 2):

1. $$\mathcal {Q}_t[i,k_T,k_S-1,s,pos] + \rho ^S_{\textsf{del}}$$
  Explanation: Intuitively, every entry $\mathcal {Q}[i,k_T,k_S,s,pos]$ defines some index $e'$ of $S'$ that is the end-point of every partial derivation in $\mathcal {D}(x^{(i)},N,y,k_T,k_S){}$. Thus, $S'[e']$ must be a part of any partial derivation $\mu \in \mathcal {D}(x^{(i)},N,y,k_T,k_S){}$, so, either $S'[e']$ is deleted under $\mu$ or it is mapped under $\mu$. The former option is captured by this case.
2. $$\displaystyle \min _{\begin{array}{c} \mu \in \mathcal {D}_\le {(x^t_{[i]},N_{x_i},x_i,k_T,k_S)} \end{array}} \displaystyle \min _{\begin{array}{c} 1 \le j \le i-1 \\ s' \in \{+,-\} \end{array}} \mathcal {Q}_t[j, k_T-\mu .del_T-\textsf{span}(x^t_{[j+1:i-1]}),k_S-\mu .del_S,s',\,\,pos-\mu .pos] + \mu .score + \delta ^Q_{\textsf{ord}}\cdot \textsf{BPDelta2}(x,x_i,x_j,N_{x_i,x_j}) + \rho ^T_{\textsf{del}}\cdot \textsf{span}(x^t_{[j+1:i-1]})$$
  Explanation: If $S'[e']$ is mapped under $\mu$, then due to the hierarchical structure of $T_x$, it must be mapped under some derivation $\mu '$ of one of the children of *x* that are in $x_{[i]}$. Thus, we receive the second case of the recursion rule. In this case, we consider $x_j \in x_{[i-1]}$ and derivations of $x_j$ only to suffixes of $S'[1:E(x_{[j]},k_T-\mu .del_T,k_S-\mu .del_S)]$.
  (2)

Once both DP tables are filled, for every combination of $(pos,k_T,k_S)$ the algorithm returns the divergence from $T_x$ to $S'$. This is calculated by going over the partial derivations, and deleting the ignored nodes (while penalizing the deletion accordingly), while considering both orders of $\textsf{children}(x)$.

Recall that we want to take into account the option of *x* to flip, and penalize accordingly. Thus, we run Algorithm 6 twice, and store the outputs for each combination $(k_T,k_S,pos)$, with small modifications in the second run. The first time we run it as it is (while ignoring the option of *x* to flip). In the second run, we capture the option of *x* to flip. We can do that by looking only at derivations where all the nodes changed there sign (if a node $y \in \textsf{children}(x)$ has sign of $+$ in $T_x$, then we consider its sign in the derivation as −, and vice versa). Specifically, we run Algorithm 6 with the following modifications. In Algorithm 6, in line 3 we define $t=r$, in line 6 we define *s* as the opposite sign of $x_i$ in $T_x$, in line 21 we define $t=r$ and *s* as the opposite sign of $x_i$ in $T_x$. In Expression 2, we define $s'$ as the opposite sign of $x_j$ in $T_x$. In addition, for every combination $(k_T,k_S,pos)$, we apply the penalty for flipping *x*, by adding $\delta ^Q_{\textsf{flip}}$ to each value. After that, we need to apply $\textsf{FlipCorrection}(x,x')$ (recall Definition 14) where $x'$ is the equivalent node that corresponds to the derivation of the entry. Note that we need to correct the flip penalties of $\textsf{children}(x)$ that are not deleted in the derivation; to do so, we can simply backtrack the values of the DP table to receive the derivation and in particular find the children who are not deleted.

Finally, we return the minimum of the two runs for every combination $(k_T,k_S,pos)$.

### Complexity analysis

In this section we analyse the time and space complexities of the algorithm that solves $\textsf{TTSD}{}$, Algorithm 4. First, we analyse Algorithms 5 and 6, which are used as procedures in Algorithm 4. For a given PQ-tree, we denote by $\gamma$ the maximum number of children of an internal node.

#### **Lemma 6.1**

*The P-Mapping algorithm, Algorithm 5, takes* $(5^\gamma m^2 n d_T^2 d_S^2 \gamma ^3)$* time and *$O(5^\gamma m d_T d_S)$* space.*

#### *Proof*

The most space consuming part of the algorithm is the 7-dimensional DP table. The first dimension, *C*, can be any subset of the set $\textsf{children}(x)$, the second and third dimensions ($C'$ and *N*) can be any subset of *C*, therefore the size of all three dimensions together is $O(5^\gamma )$ as explained next. Every choice of $C, C', N$ defines (one-to-one) a partition of $\textsf{children}(x)$ to five sets, $\textsf{children}(x) {\setminus } C, C {\setminus } (C' \cup N), C' {\setminus } N, N {\setminus } C', N \cap C'$. Thus, in order to go over all $C, C', N$, we can go over all partitions of $\textsf{children}(x)$ to five sets, which takes $O(5^{|\textsf{children}(x)|})=O(5^\gamma )$. The size of the fourth dimension (i.e. *pos*), in the worst case (where *x* is the root), is $\textsf{span}(x)=m$. The fifth dimension, *y*, can be any node in *C*, therefore the size of the dimension is $O(\gamma )$. The size of the sixth and seventh dimensions (i.e. $k_T$ and $k_S$) are $d_T+1$ and $d_S+1$, respectively. Hence, the space of the algorithm is $O(5^\gamma m \gamma d_T d_S) = O(5^\gamma m d_T d_S)$.

The algorithm has three parts: initialization, filling the DP table, and returning the derivations. The most time consuming calculation required in the initialization is the calculation of $\mathcal {D}_{(C,N,y,pos,k_T,k_S)}$ and checking if $C'$ is a vertex cover of $G[x^{(C )},x'^{(C)}]$. For a given tuple $(C,N,y,pos,k_T,k_S)$ it takes $O(|\mathcal {D}{}|)$ time to calculate the set $\mathcal {D}_{(C,N,y,pos,k_T,k_S)}$ (by naively going over each derivation in $\mathcal {D}{}$ and checking if it fits the values in the tuple); notice that $|\mathcal {D}{}|=O(m n d_T d_S)$. We calculate this set for each combination of $(C,N,y,pos,k_T,k_S)$. Thus, the calculations for $\mathcal {D}_{(C,N,y,pos,k_T,k_S)}$ take $O(2^{2\gamma } \gamma m^2 n d_T^2 d_S^2)$. In addition, we check if $C'$ is a vertex cover of $G[x^{(C )},x'^{(C)}]$. To generate $G[x^{(C )},x'^{(C)}]$ we go over all pairs of nodes in *C*, check if they changed their signed order, and if required, connect them with an edge in the graph. For a pair of node, it takes $O(\gamma )$ to check if they changed their signed order (naively). Thus it takes $O(\gamma ^3)$ to generate the graph. After that it takes $O(\gamma ^2)$ to check if $C'$ is a vertex cover (naively). Hence, the first part of the algorithm takes $O(5^\gamma m d_T d_S \gamma ^3) + O(2^{2\gamma } \gamma m^2 n d_T^2 d_S^2) = O(5^\gamma m^2 n d_T^2 d_S^2 \gamma ^3)$.

The second part of the algorithm is done by calculating the value of every entry in the $O(5^\gamma m d_T d_S)$ entries of $\mathcal {P}{}$, using the recursion rule in Expression 1. The first line among the rule takes *O*(1) time, since it involves looking in another entry of $\mathcal {P}{}$ and basic computations. The second line of the rule involves going over all derivations $\mu \in \mathcal {D}_\le {(C,N,y,k_T,k_S)}$. Namely, going over all derivations of *y* with a specific end-point, that has no more than a specific number of deletions from the tree and string, and that are consistent with *N* (i.e. $\mu .e=E(C,k_T,k_S)$, $\mu .v = y$, $\mu .del_T\le k_T$, $\mu .del_S\le k_S$ and $\mu .pos \le (\textsf{span}(y)-\mu .del_T)/2$ if $y \in N$ or $\mu .pos \ge (\textsf{span}(y)-\mu .del_T)/2$ if $y \notin N$). The number of deletions from the tree and string are bounded by $d_T$ and $d_S$, respectively, and the number of *pos* is bounded by *m*. Afterwards, we go over all $z \in C \setminus \{y\}$, whose number is bounded by $\gamma$. In addition, we use the procedures $\textsf{BPDelta}$ which can be calculated in $O(\gamma )$ time and $\textsf{JumpViolationDelta}$ which takes *O*(1) time. Thus the second line of the rule takes $O(d_T d_S m \gamma ^2)$. The third line of the rule is similar to the second line, except in addition we calculate $C_{y,z}$ and calculate the span of $C_{y,z}$, which take $O(\gamma )$ time. Thus, the third line of the rule takes $O(d_T d_S m \gamma ^2)$ time. Hence, the time to calculate one entry of $\mathcal {P}{}$ is $O(d_T d_S m \gamma ^2)$. In total, the second part of the algorithm takes $O(5^\gamma m^2 d_T^2 d_S^2 \gamma ^2)$ time.

Finally, to construct the returned set of derivations, the algorithm goes over every combination $k_T,k_S,pos$ once, i.e. it takes $O(d_T d_S m)$ time. In total, the algorithm takes $O(5^\gamma m^2 n d_T^2 d_S^2 \gamma ^3) + O(5^\gamma m^2 d_T^2 d_S^2 \gamma ^2) + (d_T d_S m) = O(5^\gamma m^2 n d_T^2 d_S^2 \gamma ^3)$ time. $\square$

#### **Lemma 6.2**

*The Q-Mapping algorithm, Algorithm 6, takes* $O(\gamma ^2 d_T^2 d_S^2\,m^2 n)$* time and *$O(\gamma d_T d_S m)$* space.*

#### *Proof*

The most space consuming part of the algorithm is the 5-dimensional DP table. The first dimension, *i*, is bounded by $\gamma$. The size of the second and third dimensions (i.e. $k_T$ and $k_S$) are $d_T+1$ and $d_S+1$, respectively. The size of the fourth dimension (i.e. $s \in \{+,-\}$) is 2. The size of the fifth dimension (i.e. *pos*), in the worst case (where *x* is the root), is $\textsf{span}(x)=m$. Hence, the space of the algorithm is $O(\gamma d_T d_S m)$.

The algorithm has three parts: initialization, filling the DP table, and returning the derivations. The most time consuming calculation required in the initialization is the calculation of $\mathcal {D}_{(x_{[i]},s,x_i,pos,k_T,k_S)}$. For a given tuple $(x_{[i]},s,x_i,pos,k_T,k_S)$ it takes $O(|\mathcal {D}{}|)$ time to calculate the set $\mathcal {D}_{(C,N,y,pos,k_T,k_S)}$ (by naively going over all derivations in $\mathcal {D}{}$ and checking if it fits the values in the tuple), notice that $|\mathcal {D}{}|=O(m n d_T d_S)$. We calculate this set for each combination of $(i,k_T,k_S,s,pos)$ where $i=1$. Thus, the calculations for $\mathcal {D}_{(x_{[i]},s,x_i,pos,k_T,k_S)}$ take $O(m^2 n d_T d_S)$ all together. Hence, the first part of the algorithm takes $O(m^2 n d_T d_S)$.

The second part of the algorithm is done by calculating the value of every entry in the $O(\gamma d_T d_S m)$ entries of $\mathcal {Q}{}$, using the recursion rule in Expression 2. The first line among the rule takes *O*(1) time, since it involves looking in another entry of $\mathcal {Q}{}$ and basic computations. The second line of the rule involves going over all derivations $\mu \in \mathcal {D}_\le {(x_{[i]},N_{x_i},x_i,k_T,k_S)}$. Namely, going over all derivations of *y* with a specific end-point, which have no more than a specific number of deletions from the tree and string, and which are consistent with $N_{x_i}$ (i.e. $\mu .e=E(x_{[i]},k_T,k_S)$, $\mu .v = x.i$, $\mu .del_T\le k_T$, $\mu .del_S\le k_S$ and $\mu .pos \le (\textsf{span}(x_i)-\mu .del_T)/2$ if $x_i \in N_{x_i}$ or $\mu .pos \ge (\textsf{span}(x_i)-\mu .del_T)/2$ if $y \notin N_{x_i}$). The number of deletions from the tree and string are bounded by $d_T$ and $d_S$, respectively, and the number of values for *pos* is bounded by *m*. Afterwards, we go over indices $1 \le j \le i-1$ and sign $s' \in \{+,-\}$, whose number is bounded by $O(\gamma )$. In addition, we use the procedure $\textsf{BPDelta2}$ which can be calculated in $O(\gamma )$ time. Thus, the second line of the rule takes $O(d_T d_S m \gamma )$. Hence, the second part of the algorithm takes $O(\gamma ^2 d_T^2 d_S^2 m^2)$ time.

Finally, to construct the returned set of derivations, the algorithm goes over every combination $k_T,k_S,pos$ once, and taking the minimum over $t \in \{\ell , r\}$, $1 \le i \le \gamma$ and $s \in \{+,-\}$; so, this takes $O(d_T d_S m \gamma ) = O(d_T d_S m)$ time. Recall that we calculate both $Q_\ell$ and $Q_r$, but this does not affect the magnitude of the time. In addition, recall that we run the algorithm twice, while in the second run there are modifications that do not increase the time (in fact, they even improve it). Hence, in total, the algorithm takes $O(m^2 n d_T d_S) + O(\gamma ^2 d_T^2 d_S^2\,m^2) + O(d_T d_S m) = O(\gamma ^2 d_T^2 d_S^2\,m^2 n)$ time. $\square$

#### **Lemma 6.3**

*The main algorithm, Algorithm 4, takes* $O(n^2 \gamma ^2 {d_T}^2 {d_S}^2\,m^2 (m_p \cdot 5^\gamma \gamma + m_q))$ *time and* $O(d_T d_S m (m n + 5^\gamma ))$ *space.*

#### *Proof*

The number of leaves in the PQ-tree *T* is *m*, hence there are *O*(*m*) nodes in the tree, i.e the size of the first dimension of the DP table, $\mathcal {A}{}$, is *O*(*m*). The size of the second dimension (i.e. *pos*), in the worst case (where *x* is the root), is $\textsf{span}(x)=m$. In the algorithm description (“The main algorithm” section) a bound for the possible start indices of subsequences derived from nodes in *T* is given (for a node *x*, the start index *i* runs between 1 and $n-(\textsf{span}(x)-d_T)+1$). The node with the largest span in *T* is the root that has a span of *m*. The root is mapped to the longest subsequence when there are $d_S$ deletions from the string. Hence, the size of the third dimension of $\mathcal {A}{}$ is $O(n-(m+d_S)+1) = O(n)$. The fourth and the fifth dimensions of $\mathcal {A}{}$ are of size $d_T+1$ and $d_S+1$, respectively. In total, the DP table $\mathcal {A}{}$ is of size $O(d_T d_S m^2 n)$.

In the initialization step, $O(d_T d_S m^2 n)$ entries of $\mathcal {A}{}$ are computed. We go over $0 \le k_S \le d_S$ and $pos \in \{0,1\}$. The most time consuming step is the generation of the set $I_{x,S,i,k_S,pos}$, and it can be generated in $O(|\textsf{children}(x)|)=O(\gamma )$ time. Thus the initialization takes $O(d_T d_S m^2 n \gamma )$. The P-Mapping algorithm is called for every P-node in *T* and every possible start index *i*, so the P-Mapping algorithm is called $O(n m_p)$ times. Similarly, the Q-Mapping algorithm is called $O(n m_q)$ times. Thus, it takes $O(d_T d_S m^2 n \gamma ) + O(n \cdot (m_p \cdot \text {Time(P-Mapping)} + m_q \cdot \text {Time(Q-Mapping)}))$ time to fill the DP table.

In the final stage of the algorithm, the minimum over the entries corresponding to every combination of ($0\le k_T\le d_T$, $0\le k_S \le d_S, 1\le i\le n-(\textsf{span}(x)-d_T)+1\}, 0 \le pos \le \textsf{span}(root_T)-k_T$) is computed. So, it takes $O(d_T d_S n m)$ time to find a derivation with minimum score.

From Lemma 6.1, the P-Mapping algorithm takes $(5^\gamma m^2 n d_T^2 d_S^2 \gamma ^3)$ time and $O(5^\gamma m d_T d_S)$ space, and from Lemma 6.2, the Q-Mapping algorithm takes $O(\gamma ^2 d_T^2 d_S^2\,m^2 n)$ time and $O(\gamma d_T d_S m)$ space. Thus, in total, our algorithm runs in $O(d_T d_S m^2 n \gamma ) + O(n \cdot (m_p \cdot O(5^\gamma m^2 n d_T^2 d_S^2 \gamma ^3) + m_q \cdot O(\gamma ^2 d_T^2 d_S^2\,m^2 n) )) = O(n^2 \gamma ^2 {d_T}^2 {d_S}^2\,m^2 (m_p \cdot 5^\gamma \gamma + m_q))$ time. Adding to the space required for the main DP table the space required for the P-Mapping algorithm (the space needed for the Q-Mapping algorithm is insignificant with respect to the P-Mapping algorithm) results in a total space complexity of $O(d_T d_S m^2 n) + O(5^\gamma m d_T d_S) = O(d_T d_S m (m n + 5^\gamma ))$. $\square$

## $\textsf{TTSD}{}$: polynomial space complexity

In this section we propose an improved version (in terms of space complexity) of the algorithm presented in “TreeToString Divergence: algorithm” section 6, using the inclusion–exclusion principle. Specifically, the space complexity of the algorithm is polynomial instead of exponential. Our P-Mapping algorithm (see “P-node mapping: the algorithm”, Algorithm 5) uses a DP table whose size is exponential. Thus, we propose a new version of the P-Mapping algorithm, which uses a DP table whose size is polynomial. In what follows, we first describe the inclusion–exclusion principle (“Inclusion–exclusion principle” section). Then, we describe the new version of the P-Mapping algorithm (“P-node mapping: polynomial space complexity” section) that is used in Algorithm 4 instead of Algorithm 5. Note that in this version, we are unable to consider jumps of large units. So, when we calculate $\textsf{Diverge}{}$, we assume that $\Delta ^P_{\textsf{jump}}{}=0$.

### Inclusion–exclusion principle

Usually, the inclusion–exclusion principle is described as a formula for computing $|\bigcup \limits _{i=1}^{n} A_i|$ for a collection of sets $A_1,\dots ,A_n$. We use the intersection version of inclusion–exclusion, which is described as a formula for computing $|\bigcap \limits _{i=1}^{n} A_i|$. Denote by [*n*] the set of indices from 1 until *n*, i.e. $[n]=\{1,\dots ,n\}$. Let $A_1,\dots ,A_n \subseteq U$, where *U* is a finite set. Denote $\bigcap _{i \in \emptyset }(U \setminus A_i)=U$. Then,

5 $$\begin{aligned} \left| \bigcap \limits _{i \in [n]} A_i \right| = \sum _{X \subseteq [n]} (-1)^{|X|}\left| \bigcap \limits _{i \in X} (U \setminus A_i) \right| . \end{aligned}$$

In typical algorithmic applications of the inclusion–exclusion formula we need to count some objects that belong to a universe *U*, and it is in some sense hard. More precisely, we are interested in objects that satisfy *n* requirements $A_1,\dots ,A_n$, and each requirement is defined as the set of objects that satisfy it. Thus, the inclusion–exclusion formula translates the problem of computing $|\bigcap \limits _{i \in [n]} A_i|$ into computing $2^n$ terms of the form $|\bigcap \limits _{i \in X} (U \setminus A_i)|$. In our application, computing these terms is in some sense easy. If, for example, each of the terms $|\bigcap \limits _{i \in X} (U {\setminus } A_i)|$ can be computed in polynomial time, then the inclusion–exclusion formula gives an algorithm that performs $2^n n^{\mathcal {O}(1)}$ arithmetic operations.

### P-node mapping: polynomial space complexity

Recall that the input consists of an internal P-node *x*, a string $S'$, bounds on the number of deletions from the tree *T* and the string $S'$, $k_T$ and $k_S$, respectively, and a set of derivations $\mathcal {D}{}$ (see Eq. 1). The output of the algorithm is the collection of divergences of derivations of *x* to every possible prefix of $S'$ having exactly $d_T$ deletions from the tree, $d_S$ deletions from the string, and *pos* leaves with positive sign, for each combination of $0 \le k_T \le d_T$, $0 \le k_S \le d_S$ and $0 \le pos \le \textsf{span}(x)$. Thus, there are $O(d_T \cdot d_S \cdot \textsf{span}(x))$ derivations in the output.

Denote by $\gamma$ the number of children of *x*, i.e. $\gamma =|\textsf{children}(x)|$. First, we define the collection of sets $A_1,\dots ,A_\gamma$ (see “Inclusion–exclusion principle” section). Notice that the derivations we seek take into account all of $\textsf{children}(x)$, using each child exactly once (mapped or deleted). Thus, we define the collection $A_1,\dots ,A_\gamma$ where $A_i$ is the set of derivations that use the *i*’th child of *x* at least once (mapped or deleted), and use exactly $\gamma$ children of *x* (including repetitions). In particular, note that the same child can be both mapped several times (to different subsrings) and deleted several times (even if it has been mapped). As a result, $\bigcap \limits _{i \in [\gamma ]} A_i$ is the set of derivations that take into account all of $\textsf{children}(x)$, where each child is used exactly once. We define *U* as the set of all partial derivations using $\gamma$ children of *x* (including repetitions). Therefore, $\bigcap \limits _{i \in X} (U \setminus A_i)$ is the set of derivation that do not use the children whose indices are in *X*, and use exactly $\gamma$ children of *x* (including repetitions).

Denote by *ScoreSet* the set of all possible divergence values of all partial derivations of *x* to $S'$. We use the collection of sets $A_1,\dots ,A_\gamma$ as described above, for every combination of $(k_T,k_S,pos,score)$ where the divergence values of the derivations in $A_1,\dots ,A_\gamma$ are at most *score*. Specifically, Algorithm 7 constructs a 4-dimensional DP table $\mathcal {B}{}$, which has an entry for every $0\le k_T \le d_T$, $0\le k_S \le d_S$, $0 \le pos \le \textsf{span}(x)$ and $score \in ScoreSet$. The purpose of an entry $\mathcal {B}[k_T,k_S,pos,score]$ is to hold the number of partial derivations rooted in *x* to a prefix of $S'$ with exactly $k_T$ deletions from the tree, $k_S$ deletions from the string, *pos* leaves with positive sign in the derivation whose divergence value is at most *score*. For each entry in the DP table, we calculate its value using the intersection version of the inclusion–exclusion formula, shown in “Inclusion–exclusion principle” section.

#### **Lemma 7.1**

*Assuming that Algorithm 8 is correct,*^6^* Algorithm 7 is correct, that is, it returns the collection of divergences of derivations of x* to every prefix of $S'$* having exactly* $k_T$* deletions from* $T_x$, $k_S$* deletions from the prefix of *$S'$ *and pos number of leaves with positive sign, for each combination of *$0 \le k_T \le d_T$, $0 \le k_S \le d_S$* and* $0 \le pos \le \textsf{span}(x)$.

#### *Proof*

For each combination of $0 \le k_T \le d_T$, $0 \le k_S \le d_S$, $0 \le pos \le \textsf{span}(x)$ and $score \in ScoreSet$, we define $\hat{U} \subseteq U$ as the set of all partial derivations using $\gamma$ children of *x* (including repetitions) with exactly $k_T$ deletions from the tree, $k_S$ deletions from the string, *pos* leaves with positive sign in the derivation whose divergence value is at most *score*. We define $\hat{A_i} \subseteq \hat{U}$ as the set of derivations that use the *i*’th child of *x* at least once (mapped or deleted), and use exactly $\gamma$ children of *x* (including repetitions). For each combination $(k_T, k_S, pos, score)$, $\bigcap \limits _{i \in [\gamma ]} \hat{A_i}$ is the set of derivations with exactly $k_T$ deletions from the tree, $k_S$ deletions from the string, *pos* leaves with positive sign in the derivation whose divergence value is at most *score* that take into account all of $\textsf{children}(x)$, where each child is used exactly once. For each combination $(k_T, k_S, pos, score)$, and given $Y \subseteq [\gamma ]$, Algorithm 8 returns $|\bigcap \limits _{i \in Y} (\hat{U} \setminus \hat{A_i})|$, which is the number of derivations that do not use the children whose indices are in *Y*, and use exactly $\gamma$ children of *x* (including repetitions). In Algorithm 7 we calculate $|\bigcap \limits _{i \in [\gamma ]} \hat{A_i}|$ for each combination $(k_T, k_S, pos, score)$ according to the Inclusion–Exclusion principle and store the results in table $\mathcal {B}{}$, using Eq. 5 as follows.

$$\begin{aligned} \left| \bigcap \limits _{i \in [\gamma ]} \hat{A_i} \right| = \sum _{Y \subseteq [\gamma ]} (-1)^{|Y|} \left| \bigcap \limits _{i \in Y} (\hat{U} \setminus \hat{A_i}) \right| . \end{aligned}$$

From the correctness of Algorithm 8 and the correctness of Eq. 5, the values in $\mathcal {B}{}$ are correct.
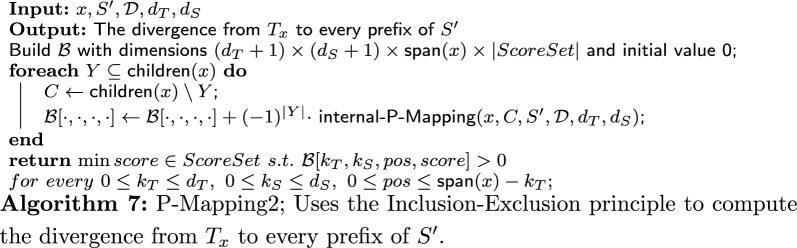


Finally, for each combination of $0 \le k_T \le d_T$, $0 \le k_S \le d_S$ and $0 \le pos \le \textsf{span}(x)$, Algorithm 7 returns the minimum $score \in ScoreSet$ for which $\mathcal {B}[k_T,k_S,pos,score]>0$, meaning that *score* is the minimum value of the possible derivations, that is, the desired divergence value to be returned.

$$\square $$

### Internal P-node mapping

In this section, we describe Algorithm 8. The input consists of an internal P-node *x*, a subset $C \subseteq \textsf{children}(x)$, a string $S'$, bounds on the number of deletions from the tree *T* and the string $S'$, $d_T$ and $d_S$, respectively, and a set of derivations $\mathcal {D}{}$ (see Eq. 1). The output of the algorithm is the number of all partial derivations of $x^{(C)}$ to every prefix of $S'$ for each combination $(k_T,k_s,pos,score)$ having exactly $k_T$ deletions from the tree, $k_S$ deletions from the string, *pos* leaves with positive sign, whose divergence value is at most *score*, and that use $\gamma$ children from *C* (including repetitions). Thus, there are $O(d_T \cdot d_S \cdot \textsf{span}(x) \cdot |ScoreSet|)$ derivations in the output.

Algorithm 8 constructs an 8-dimensional DP table $\mathcal {P}{}$, which has an entry for every $1\le i \le |\textsf{children}(x)|$, $y \in C$, $s \in \{+,-\}$, $1\le j \le |S'|$, $0 \le pos \le \textsf{span}(x)$, $0\le k_T \le d_T$, $0\le k_S \le d_S$, and for every $score \in ScoreSet$. The purpose of an entry $\mathcal {P}[i,y,s,j,pos,k_T,k_S,score]$ is to hold the number of all partial derivations rooted in $x^{(C )}$ to a suffix of $S'[1:j]$, which use exactly *i* children from *C* (not necessarily different children), which map *y* to the suffix of $S'[1:j]$, with exactly $k_T$ deletions from the tree, $k_S$ deletions from the string, *pos* leaves with positive sign in $T_{y'}$ such that *s* is the sign of *y* in the derivation and it is consistent with *pos*, and whose divergence value is at most *score*.

Every entry $\mathcal {P}[i,y,s,j,pos,k_T,k_S,score]$ for which $i=1$ is initialized with $|\mathcal {D}_{(i,y,s,j,pos, k_T,k_S,score)}|$, which is the size of the set of all partial derivations rooted in $T_y$ to a suffix of $S'[1:j]$, with exactly $k_T$ deletions from the tree, $k_S$ deletions from the string, *pos* leaves with positive sign in $T_{y'}$ such that *s* is the sign of *y* in the derivation and it is consistent with *pos*, and whose divergence value is at most *score*.

After the initialization, the remaining entries of $\mathcal {P}{}$ are calculated using the recursion rule in Expression 3 ahead. The order of computation is ascending with respect to the number of children that should be used *i*, and it is also ascending with respect to the number of deletions from both tree and string. In addition, the order is ascending with respect to the number of positive signed leaves in the derivation, and it is also ascending with respect to the maximum score of the derivations.
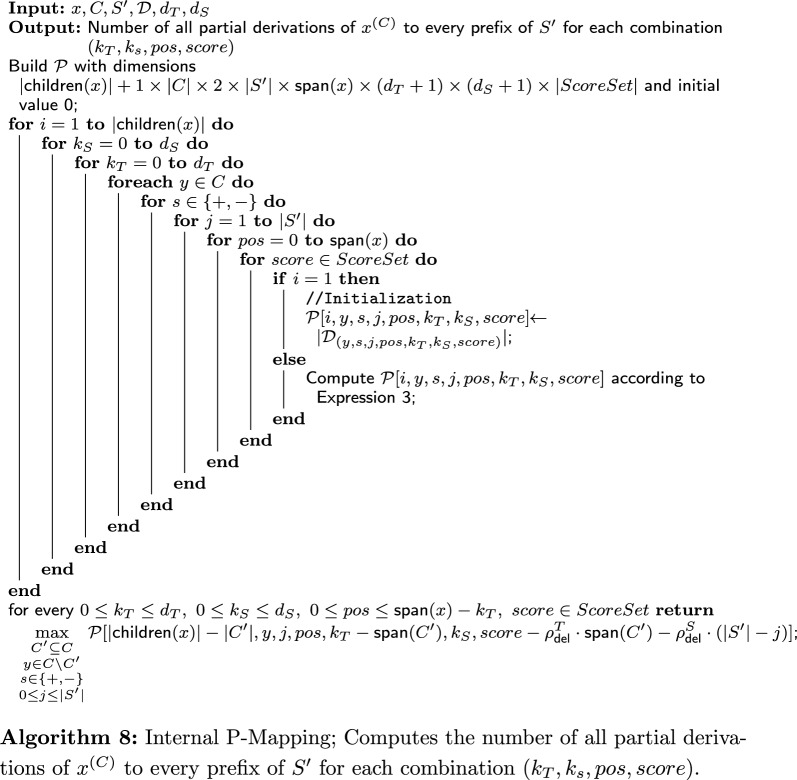


In Expression 3 we use the notations $N_{y}$ and $N_{y,z}$, defined as follows. Let *y* and *z* be two nodes where the signs of them in $T_{x'^{(i)}}$ considered to be *s* and $s'$ respectively. If $s=-$ then $y \in N_{y}$ and $y \in N_{y,z}$, and if $s'=-$ then $z \in N_{y,z}$. If $s=+$ then $y \notin N_{y}$ and $y \notin N_{y,z}$, and if $s'=+$ then $z \notin N_{y,z}$. In addition, we use the notation $\mathcal {D}_\le {(j,N_{y},y,k_T,k_S)}$ instead of $\mathcal {D}_\le {(C,N_{y},y,k_T,k_S)}$ (see Definition 22). The two sets are similar except that the derivations in $\mathcal {D}_\le {(j,N_{y},y,k_T,k_S)}$ are derivations of *y* to a suffixes of $S'[1:j]$.

In the algorithm, $\mathcal {P}[i,y,s,j,pos,k_T,k_S,score]$ is computed by taking the minimum between the following expressions (we will refer the minimum of the three expressions as Expression 3):

1. $$\mathcal {P}[i,y,s,j-1,pos,k_T,k_S-1,score-\rho ^S_{\textsf{del}}]$$
  Explanation: For every entry $\mathcal {P}[i,y,s,j,pos,k_T,k_S,score]$, *j* is the end-point of every partial derivation. Thus, $S'[j]$ must be a part of any partial derivation; so, $S'[j]$ is either deleted or mapped. The former option is captured by the first case of the recursion rule.
2. $$\displaystyle \sum _{\begin{array}{c} z \in {C} \\ s' \in \{+,-\} \end{array}} \mathcal {P}[i-1,z,s',j-\textsf{span}(y)+\mu .del_T,pos-\mu .pos,k_T-\mu .del_T,\,k_S-\mu .del_S, score - \mu .score - \textsf{BPDelta}(x,y,z,N_{y,z})]$$
  Explanation: If $S'[j]$ is mapped, then due to the hierarchical structure of $T_x$, it must be mapped under some derivation $\mu '$ of one of the children of *x* that are in *C*. Thus, we receive the second and the third cases of the recursion rule. In these cases we take into account every $z \in C$ to be aligned to the suffix of the derivation’s subsequence $S'[1:j-\textsf{span}(y)+\mu .del_T]$.
3. $$\displaystyle \sum _{\begin{array}{c} z \in {C} s.t. C_{y,z}\subseteq C \\ s' \in \{+,-\} \end{array}} \mathcal {P}[i - |C_{y,z}| - 1,z,s',j-\textsf{span}(y) \! + \! \mu .del_T,pos \! - \! \mu .pos,\,k_T \! - \! \mu .del_T\! - \!\textsf{span}(C_{y,z}), k_S-\mu .del_S, score - \mu .score- \rho ^T_{\textsf{del}}\cdot \textsf{span}(C_{y,z}) - \textsf{BPDelta2}(x,y,z,N_{y,z})]$$
  Explanation: The third case captures the option of deleting all the nodes between *y* and *z* ($C_{y,z}$), so that after the deletion we consider *y* and *z* as adjacent in $T_{x^{(C )}}$.
  (3)

Once the entire DP table is filled, for every combination of $(pos,k_T,k_S,score)$ the algorithm returns the number of all partial derivations of $x^{(C)}$ to every prefix of $S'$, which is calculated by going over the the values of the DP table and now delete the ignored nodes and penalize the deletion ($C' \subseteq C$ is the set of children considered to be deleted).

### Complexity analysis

In this section we analyse the time and space complexities of the improved algorithm that solves $\textsf{TTSD}{}$, Algorithm 4, which uses the improved P-Mapping algorithm. First, we analyse Algorithms 8 and 7, which are used as sub procedures in Algorithm 4 (instead of Algorithm 5).

#### **Lemma 7.2**

*The Internal P-Mapping algorithm, Algorithm 8, takes* $O(2^\gamma \gamma ^4\,m^2 n^2 d_T^2 d_S^2 (d_T+d_S+m+n))$* time and *$O(\gamma ^2 n m d_T d_S (d_T+d_S+m+n))$* space.*

#### *Proof*

The most space consuming part of the algorithm is the 8-dimensional DP table. The first dimension, $1 \le i \le |\textsf{children}(x)|$, is of size $O(|\textsf{children}(x)|)=O(\gamma )$. The the second dimention, *y*, can be any node in *C*, therefore the size of the second dimension is $O(|C|)=O(|\textsf{children}(x)|)=O(\gamma )$. The third dimension, $s\in \{+,-\}$, is of size 2. The fourth dimension, $1 \le j \le |S'|$, is of size *O*(*n*). The size of the fifth dimension (i.e. *pos*), in the worst case (where *x* is the root), is $\textsf{span}(x)=m$. The size of the sixth and seventh dimensions (i.e. $k_T$ and $k_S$) are $d_T+1$ and $d_S+1$, respectively. The eighth dimension, *score*, can be any score in *ScoreSet*. For fixed penalty parameters $\delta ^Q_{\textsf{ord}}$ and $\delta ^Q_{\textsf{flip}}$, in the worst case, we pay the maximum number of possible break-point (bounded by $m+n$), pay the maximum number of deletions from the tree and string $d_T+d_S$, and pay for a flip of all nodes in the tree (which yields *m* values). Therefore the size of the eighth dimension is $O(|ScoreSet|)=O(d_T+d_S+m+n)$. Hence, the space of the algorithm is $O(\gamma ^2 n m d_T d_S (d_T+d_S+m+n))$.

The algorithm has three parts: initialization, filling the DP table, and returning the derivations. The most time consuming calculation required in the initialization is the calculation of $\mathcal {D}_{(y,s,j,pos,k_T,k_S,score)}$. For a given tuple $(i,y,s,j,pos,k_T,k_S,score)$ it takes $O(|\mathcal {D}{}|)$ time to calculate the set $\mathcal {D}_{(y,s,j,pos,k_T,k_S,score)}$ (by naively going over all derivations in $\mathcal {D}{}$ and checking if they fit the values in the tuple); notice that $|\mathcal {D}{}|=O(m n d_T d_S)$. We calculate this set for each combination of $(i,y,s,j,pos,k_T,k_S,score)$. Thus, the first part of the algorithm takes $O(\gamma n^2\,m^2 d^2_T d^2_S (d_T+d_S+m+n))$. The second part of the algorithm is done by calculating the value of every entry in the $O(\gamma ^2 n m d_T d_S (d_T+d_S+m+n))$ entries of $\mathcal {P}{}$, using the recursion rule in Expression 3. The first line of the rule takes *O*(1) time, since it involves looking in another entry of $\mathcal {P}{}$ and basic computations. The second and the third lines of the rule involves going over all derivations $\mu \in \mathcal {D}_\le {(j,N_y,y,k_T,k_S)}$. Namely, going over all derivations of *y* with a specific end-point, that have no more than a specific number of deletions from the tree and string, and that are consistent with *N* (i.e. $\mu .e=j$, $\mu .v = y$, $\mu .del_T\le k_T$, $\mu .del_S\le k_S$ and $\mu .pos \le (\textsf{span}(y)-\mu .del_T)/2$ if $y \in N$ or $\mu .pos \ge (\textsf{span}(y)-\mu .del_T)/2$ if $y \notin N$). The number of deletions from the tree and string are bounded by $d_T$ and $d_S$, respectively, and the number of values of *pos* is bounded by *m*. Afterwards, we go over $z \in C$, whose number is bounded by $\gamma$, and we go over $s'\in \{+,-\}$. In addition, we use the procedures $\textsf{BPDelta}$ and $\textsf{BPDelta2}$, which can be calculated in $O(\gamma )$ time. Thus the second and third lines of the rule take $O(d_T d_S m \gamma ^2)$. Hence, the time to calculate one entry of $\mathcal {P}{}$ is $O(d_T d_S m \gamma ^2)$. In total, the second part of the algorithm takes $O(\gamma ^4 n m^2 d_T^2 d_S^2 (d_T+d_S+m+n))$ time. Finally, to construct the returned set of derivations, we go over every combination $k_T,k_S,pos,score$, and for every combination, we take the maximum over $C' \subseteq C, \ y \in C, \ s \in \{+,-\}, \ 0 \le j \le |S'|$, i.e. it takes $O(2^\gamma \gamma d_T d_S m n (d_T+d_S+m+n))$ time. In total, the algorithm takes $O(\gamma n^2\,m^2 d^2_T d^2_S (d_T+d_S+m+n)) + O(\gamma ^4 n m^2 d_T^2 d_S^2 (d_T+d_S+m+n)) + O(2^\gamma \gamma d_T d_S m n (d_T+d_S+m+n)) = O(2^\gamma \gamma ^4\,m^2 n^2 d_T^2 d_S^2 (d_T+d_S+m+n))$ time. $\square$

#### **Lemma 7.3**

*The P-Mapping2 algorithm, Algorithm 7, takes* $O(2^{2\gamma } \gamma ^4 m^2 n^2 d_T^2 d_S^2 (d_T+d_S+m+n))$* time and *$O(\gamma ^2 n m d_T d_S (d_T+d_S+m+n))$* space.*

#### *Proof*

The most space consuming part of the algorithm is the 4-dimensional table. The size of the first and second dimensions (i.e. $k_T$ and $k_S$) are $d_T+1$ and $d_S+1$, respectively. The size of the third dimension (i.e. *pos*), in the worst case (where *x* is the root), is $\textsf{span}(x)=m$. The fourth dimension, *score*, can be any score in *ScoreSet*, therefore the size of the dimension is $O(|ScoreSet|)=O(d_T+d_S+m+n)$. In total, the DP table $\mathcal {B}{}$ is of size $O(d_T d_S m (d_T+d_S+m+n))$.

The algorithm has two parts: filling the table, and returning the derivations. In the first part of the algorithm, we go over every subset of $\textsf{children}(x)$, $Y \subseteq \textsf{children}(x)$, and for each one we call Algorithm 8. Thus we call Algorithm 8 $O(2^{|\textsf{children}(x)|})=O(2^{\gamma })$ times. In the second part of the algorithm, after the table is full, for every $0\le k_T\le d_T, \ 0\le k_S\le d_S, \ 0 \le pos \le \textsf{span}(x) - k_T$, we return the minimum over $score \in ScoreSet$ such that the value of the corresponding entry of the table is positive. Hence, the second part of the algorithm takes $O(d_T d_S m (d_T+d_S+m+n))$ time.

From Lemma 7.2, the Internal P-Mapping algorithm takes $O(2^\gamma \gamma ^4\,m^2 n^2 d_T^2 d_S^2 (d_T+d_S+m+n))$ time and $O(\gamma ^2 n m d_T d_S (d_T+d_S+m+n))$ space. Thus, in total, our algorithm runs in $O(2^{\gamma } 2^\gamma \gamma ^4\,m^2 n^2 d_T^2 d_S^2 (d_T+d_S+m+n)) = O(2^{2\gamma } \gamma ^4\,m^2 n^2 d_T^2 d_S^2 (d_T+d_S+m+n))$ time. Adding to the space required for table the space required for the P-Mapping2 algorithm results in a total space complexity of $O(d_T d_S m (d_T+d_S+m+n)) + O(\gamma ^2 n m d_T d_S (d_T+d_S+m+n)) = O(\gamma ^2 n m d_T d_S (d_T+d_S+m+n))$. $\square$

#### **Lemma 7.4**

*The main algorithm, Algorithm 4, using the improved P-Mapping algorithm, takes* $O(n \gamma ^2 {d_T}^2 {d_S}^2\,m^2 (m_p \cdot 2^{2\gamma }\gamma ^2n(d_T+d_S+m+n) + m_q))$* time and* $O(\gamma ^2 n m^2 d_T d_S (d_T+d_S+m+n))$* space.*

#### *Proof*

The number of leaves in the PQ-tree *T* is *m*, hence there are *O*(*m*) nodes in the tree, i.e the size of the first dimension of the DP table, $\mathcal {A}{}$, is *O*(*m*). The size of the second dimension (i.e. *pos*), in the worst case (where *x* is the root), is $\textsf{span}(x)=m$. In the algorithm description (“The main algorithm” section) a bound for the possible start indices of subsequences derived from nodes in *T* is given (for a node *x*, the start index *i* runs between 1 and $n-(\textsf{span}{x}-d_T)+1$). The node with the largest span in *T* is the root that has a span of *m*. The root is mapped to the longest subsequence when there are $d_S$ deletions from the string. Hence, the size of the third dimension of $\mathcal {A}{}$ is $O(n-(m+d_S)+1) = O(n)$. The fourth and the fifth dimensions of $\mathcal {A}{}$ are of size $d_T+1$ and $d_S+1$, respectively. In total, the DP table $\mathcal {A}{}$ is of size $O(d_T d_S m^2 n)$.

In the initialization step $O(d_T d_S m^2 n)$ entries of $\mathcal {A}{}$ are computed. We go over $0 \le k_S \le d_S$ and $pos \in \{0,1\}$. The most time consuming is the generation of the set $I_{x,S,i,k_S,pos}$, and can be generated in $O(|\textsf{children}(x)|)=O(\gamma )$ time. Thus the initialization takes $O(d_T d_S m^2 n \gamma )$. The improved P-Mapping algorithm is called for every P-node in *T* and every possible start index *i*, i.e. the P-Mapping algorithm is called $O(n m_p)$ times. Similarly, the Q-Mapping algorithm is called $O(n m_q)$ times. Thus, it takes $O(d_T d_S m^2 n \gamma ) + O(n\ (m_p \cdot \text {Time(P-Mapping)} + m_q \cdot \text {Time(Q-Mapping)}))$ time to fill the DP table. In the final stage of the algorithm, the minimum over the entries corresponding to every combination of ($0\le k_T\le d_T$, $0\le k_S \le d_S, 1\le i\le n-(\textsf{span}(x)-d_T)+1\}, 0 \le pos \le \textsf{span}(root_T)-k_T$) is computed. So, it takes $O(d_T d_S n m)$ time to find a derivation with minimum score.

From Lemma 7.3, the P-Mapping2 algorithm takes $O(2^{2\gamma } \gamma ^4 m^2 n^2 d_T^2 d_S^2 (d_T+d_S+m+n))$ time and $O(\gamma ^2 n m d_T d_S (d_T+d_S+m+n))$ space, and from Lemma 6.2, the Q-Mapping algorithm takes $O(\gamma ^2 d_T^2 d_S^2\,m^2)$ time and $O(\gamma d_T d_S m)$ space. Thus, in total, our algorithm runs in $O(d_T d_S m^2 n \gamma ) + O(n (m_p \cdot O(2^{2\gamma } \gamma ^4\,m^2 n^2 d_T^2 d_S^2 (d_T+d_S+m+n)) + m_q \cdot O(\gamma ^2 d_T^2 d_S^2\,m^2) )) = O(n \gamma ^2 {d_T}^2 {d_S}^2\,m^2 (m_p \cdot 2^{2\gamma }\gamma ^2n(d_T+d_S+m+n) + m_q))$ time. Adding to the space required for the main DP table the space required for the P-Mapping2 and Q-Mapping algorithms, results in a total space complexity of $O(d_T d_S m^2 n) + O(\gamma ^2 n m d_T d_S (d_T+d_S+m+n)) + O(\gamma d_T d_S m) = O(\gamma ^2 n m^2 d_T d_S (d_T+d_S+m+n))$. $\square$

## Methods and datasets

### Dataset and gene cluster generation

1487 fully sequenced prokaryotic strains with COG ID annotations were downloaded from GenBank (NCBI; ver 10/2012). The gene clusters were generated from this data using the tool CSBFinder-S [45]. CSBFinder-S was applied to all the genomes in the dataset after removing their plasmids, using parameters $q=10$ (a colinear gene cluster is required to appear in at least ten genomes) and $k=0$ (no insertions are allowed in a colinear gene cluster), resulting in 79,017 colinear gene orders. From these gene orders, only gene orders whose number of distinct COGs is between 4 and 9 were kept, leaving 28,537 gene orders. Next, ignoring strand and gene order information, colinear gene orders that contain the exact same COGs were united to form the generalized set of 91 gene clusters that abide by the requirements that each gene cluster contains at least 3 gene orders and each COG appears only once in each gene order. For each gene cluster, the most abundant gene order was designated as the “reference” (centroid) gene order. Based on this, the clusters were further filtered to keep only 63 gene clusters whose designated reference has instances in at least 30 genomes. Finally, clusters containing one or more gene orders that are identical to the designated reference gene order, in terms of the list of classes in which they have instances, were removed, leaving a benchmark set of 59 gene clusters.

### PQ-tree construction

The input PQ-trees for our algorithm where constructed using the tool PQFinder (available on GitHub [46]). PQFinder was applied to each of the gene clusters in the dataset, to build the PQ-tree representing each cluster. In addition, each Q-node with exactly two children, whose height in the tree is greater than 1, was changed to a P-node (in this special case all children of the node were observed in all shuffling options, which in our opinion better fits the syntax of a P-node than that of a Q-node.)

### Parameter settings

In our experiment, we set the parameters of the algorithm as follows. $\delta ^Q_{\textsf{ord}}=1.5$, $\delta ^Q_{\textsf{flip}}=0.5$, $d_T=0$, $d_S=0$. The stronger penalty for $\delta ^Q_{\textsf{ord}}$ versus $\delta ^Q_{\textsf{flip}}$ is based on the observation that gene clusters in prokaryotes are very strongly colinearly conserved [30, 31], even when the benchmark dataset is large and spans a wide taxonomical range of prokaryotes [32].

### Strand information

Our approach to the comparison of two gene orders focuses on adaptive fitness (in terms of the order by which the gene products are produced). Therefore, we do not distinguish between two gene orders that appear in distinct strands, however are identical in terms of the order and direction of their genes (with respect to the transcription start site). Furthermore, the tool by which the gene orders were identified (CSBfinder [45]) groups together instances from distinct strands into the same CSB. To this end, for any two gene orders being compared in our benchmarks, we compute the rearrangement distance twice: in the first computation, both gene orders preserve the original strand, and in the second computation one of the compared gene order is modified by reversing both the order and the directions of its genes.

## Results

### Evaluation

In this section we evaluate the accuracy of our approach in measuring the evolutionary divergence between two gene orders that belong to the same gene cluster. To this end, we aim to generate a set of “control” distances, computed from real data, against which the divergence scores computed by our tool can be compared and evaluated.

Recall that in our application, each of the input sequences does not correspond to a specific genomic sequence but rather represents a gene order that occurs in multiple genomes. In addition, abundant gene clusters typically display several paralogous occurrences of distinct gene orders, and possibly several paralogous occurrences of a specific gene order, within the same genome. Furthermore, each distinct occurrence of a specific gene order could differ substantially from another occurrence of the same gene order in terms of its encoding genomic sequence, since each COG represents a cluster of genomic sequences that are not identical however are similar enough (possibly based on local sequence similarity) to be clustered to the same gene orthology group. This poses a challenge in creating a set of “control” distances by which to evaluate the performance of our approach in comparison to extant genome rearrangement distances, since the “ground truth” regarding the evolutionary distance between two gene orders can not be estimated by computing the sequence alignment between the underlying genomic sequences.

Thus, we chose to represent each gene order by the assemblage of its instances, i.e. the set of genomes in which it occurs, and to employ comparative assemblage analysis as a “control” measure. Several similarity (or overlap) indices based on presence/absence (incidence) data have been proposed in the literature [47, 48]. A classical and widely used index in comparative assemblage analysis is the Jaccard index [48]. In our comparative evaluation the instance assemblages are used to estimate divergence rather than similarity, and therefore we use the inverse Jaccard Index as an estimator of the instance assemblage based divergence between two gene orders.

Our proposed divergence measure was evaluated, per each cluster, as follows: first, we applied our approach (Algorithm 1) to measure the structure informed divergence from the cluster’s designated reference (explained in “Methods and datasets” section) to each of the other gene orders. Then, we calculated the Inverse Jaccard based distance from the set of instances of the reference gene order to the sets of instances of each of the other gene orders. In order to tolerate the noise due to inter-specie and inter-genus horizontal transfer of gene orders, we first converted the assemblages of genomes to the assemblages of (taxonomic) classes to which these genomes belong. This resulted in two series of scores, which were then subjected to the computation of Spearman and Pearson correlations between them. The same evaluation procedure was then repeated twice, once using the signed break-point distance (as in Definition 8) instead of our structure-informed divergence measure, and once using the CREx reversal distance [28].

To this end, the 59 gene clusters were distributed to three groups according to the number of Q-nodes in their representative PQ-trees, as we consider the number of Q-nodes in a PQ-tree to be a good estimate of the hierarchical complexity of its colinear components. This yielded 8 gene clusters whose representative tree has no Q-nodes, 41 gene clusters whose representative tree has one Q-node, and 10 gene clusters whose representative tree has two or more Q-nodes. For each group, the average Spearman and Pearson correlation scores were computed for each of the three compared measures. The results are summarized in Table 1. Additional details regarding the comparative results per each gene cluster, as well as the functional categories of the genes in the cluster, are given in Tables 2 and 3. Note that the rows of Table 2 are sorted by decreasing value of the difference $\textsf{Diverge}-\mathsf {d_{reversals}}$, and the first result from the table is interpreted in detail in “A motivating example” section.

**Table 1:** A comparison between our proposed rearrangement measure ($\textsf{Diverge}$), signed break-point distance ($\mathsf {d_{SBP}}$) as in Definition 8, and the CREx reversals distance ($d_{reversals}$) [28], based on their correlation to a taxonomical instance abundance measure

Num of Q-nodes	Correlation	$\textsf{Diverge}$	$\mathsf {d_{SBP}}$	$\mathsf {d_{reversals}}$
0	Pearson	0.767	0.777	0.824
	Spearman	0.680	0.673	0.647
1	Pearson	0.883	0.869	0.845
	Spearman	0.775	0.753	0.697
2	Pearson	0.927	0.859	0.842
	Spearman	0.907	0.845	0.823

Table 1 indicates that, in general, the rearrangement-based divergence between a reference gene order and its target gene orders correlates well with the divergence in the taxonomic distribution of their corresponding instances, which is a very interesting result on its own, supporting our choice of gene order instance assemblage-based distance as a control measure for our comparative analysis.

**Fig. 11:**
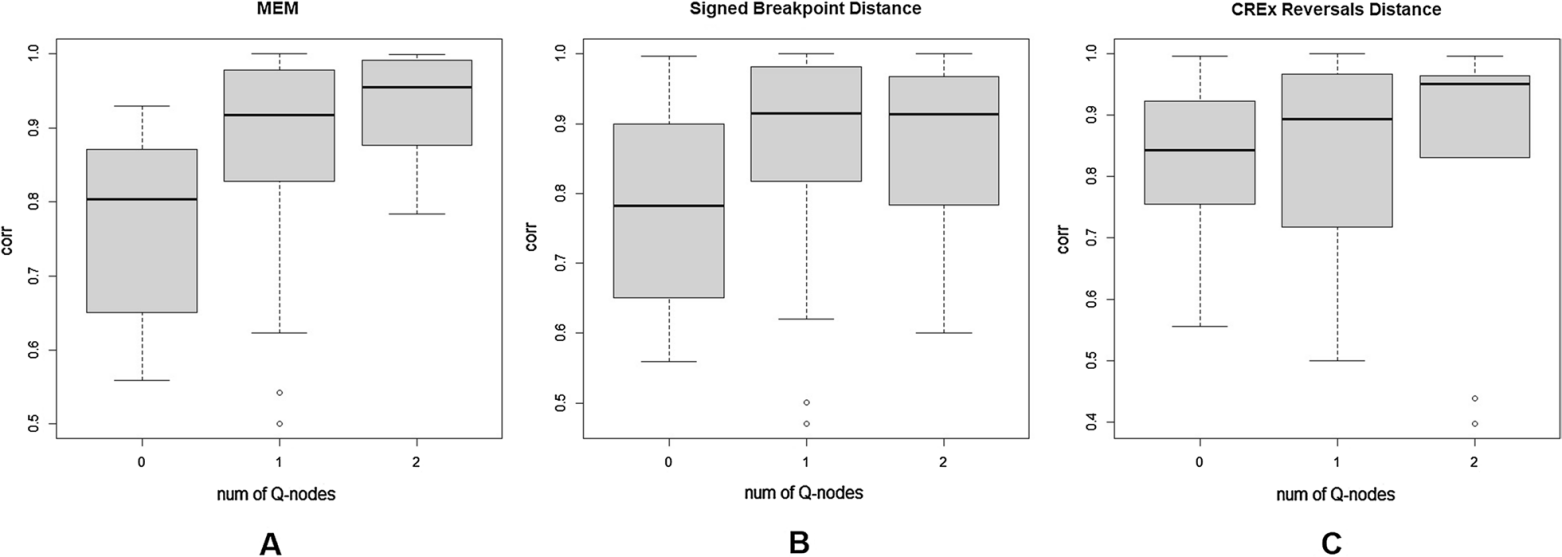
Distributions of Pearson correlations computed between a series of genomic rearrangement scores and the corresponding series of instance abundance indexes. **A** Our proposed rearrangement measure ($\textsf{Diverge}$). **B** Signed break-point distance ($\mathsf {d_{SBP}}$) as in Definition 8. **C** CREx reversals distance ($d_{reversals}$) [28]. For each measure in **A–C**, the distribution is computed and shown separately per each gene cluster group, as described above: “corr” (Y-axis) denotes the Pearson correlation, “num of Q-nodes” denotes the number of Q-nodes in the PQ-trees of the gene clusters belonging to the specific group

As expected, in gene clusters where there is very weakly conserved structure or none at all, the results are comparable to the other measures. However, the table shows that as the conserved structure of the gene cluster increases, so does our advantage over the signed break-point distance and the CREx reversal distance. In Fig. 11 we further analyse the affect of the level of colinear component co-dependency on the correlation results. Interestingly, a Kruskal-Wallis test indicates significant difference between the correlations computed for our proposed measure $\textsf{Diverge}$ for the three groups of gene clusters ($\chi ^2(2)=7.537$, P-val$=0.023$), while such is not the for the other compared measures, $\mathsf {d_{SBP}}$ ($\chi ^2(2)=2.599$, P-val$=0.273$) and $\mathsf {d_{reversals}}$ ($\chi ^2(2)=0.981$, P-val$=0.612$).

Our implementation of the first algorithm took 0.72 s to complete the analysis of the full dataset when run on a laptop with an Intel(R) Core(TM) i7-8550U CPU (1.99 GHz) using 8 GB RAM. Our implementation of the second algorithm took 5.4 min to complete the same analysis on the same laptop.

## Conclusions

In this paper, we defined two (genome rearrangement-based) problems in comparative genomics, denoted $\textsf{TTSD}{}$ ($\mathsf {TreeToString \ Divergence}$) and $\textsf{CTTSD}{}$ ($\textsf{Constrained}$ $\textsf{TTSD}{}$), where the second problem is a special case of the first one. Both problems take as input two sequences of genes $S_1$ and $S_2$, a PQ-tree *T* representing the known gene orders of a gene cluster of interest, with its leaves ordered according to sequence $S_1$. $\textsf{TTSD}{}$ also takes as input integer arguments $d_T$ and $d_S$ (we assume that in $\textsf{CTTSD}{}$, $d_T=d_S=0$). The objective is to reorder *T* as a subsequence $S_2'$ of $S_2$, allowing up to $d_T$ deletions of leaves from *T*, and such that $S_2'$ is obtained from $S_2$ by using up to $d_S$ deletions from $S_2$, while calculating a corresponding score that serves as the objective divergence measure.

We proposed an algorithm that solves $\textsf{CTTSD}$ in $O^*(1.381^{\gamma })$ time and $O^*(1)$ space. The parameter $\gamma$ is the maximum degree of a P-node in *T* and $O^*$ is used to hide polynomial factors in the input size. In the special case where the jump penalty is set to 0, the time complexity of our proposed algorithm is $O^*(1)$. In addition, we proposed a parameterized algorithm that solves $\textsf{TTSD}$ in $O^*(5^{\gamma })$ time and $O^*(5^{\gamma })$ space. Lastly, we proposed a parameterized algorithm that solves a variant of $\textsf{TTSD}$, where the jump penalty is set to 0, that reduces the space complexity of the prior algorithm, using the inclusion–exclusion principle. The algorithm take $O^*(4^{\gamma })$ time and $O^*(1)$ space.

The proposed general algorithm was implemented as a software tool and applied to the comparative and evolutionary analysis of 59 chromosomal gene clusters extracted from a dataset of 1487 prokaryotic genomes. Our preliminary results, based on the analysis of the 59 gene clusters, indicate that our proposed measure correlates well with an instance-abundance index that is computed by comparing the class composition of the genomic instances of two compared gene orders. Comparative analysis versus two extant methods (using very preliminary and simple scoring parameter values for our proposed engine) yields equivalent results in terms of the average correlations to the instance-abundance index values obtained for the benchmark dataset. In future work we propose to assemble a large dataset of prokaryotic gene clusters (large enough to train a more sophisticated scoring scheme model) and use it to train our scoring scheme parameters.

Our proposed measure, however, is shown to more sensitively capture and utilize the conserved structural information characterizing a gene cluster: The statistical test we conducted indicates that as the structural information of a gene cluster, as encoded by its PQ-tree, increases - our proposed measure significantly improves in terms of computing rearrangement scenarios between gene orders belonging to the cluster. This, as opposed to the other two extant measures tested the experiment.

One of the downsides of using PQ-trees to represent gene clusters is that very rare gene orders taken into account in the tree construction could greatly increase the number of allowed rearrangements and thus substantially lower the specificity of the PQ-tree. Thus, a natural continuation of our research would be to increase the specificity of the model by considering a stochastic variation of the algorithms presented in this paper, or alternatively to identify and exclude gene order outliers during PQ-tree construction. In addition, future extensions of this work could also aim to increase the sensitivity of the model by taking into account gene duplications (or at least tandem duplications) and gene-fusion events, which are typical events in gene cluster evolution.

## Data Availability

The code for our tool, the data used in the experiments, and the log file produced by the run of the reported benchmark, can be found on GitHub: http://www.github.com/edenozery/MEM-Rearrange.

## Appendix

See Tables 2, 3, 4.

**Table 2:** 59 gene clusters analyzed in our experiment

#	Gene cluster ID	$\textsf{Diverge}$	$\mathsf {d_{SBP}}$	$d_{reversals}$	Size	PQ-tree
1	19876	1.000	0.655	0.500	3	((0− [1− 2− 3−]) 4+)
2	21344	0.905	0.615	0.439	4	([0− 1−] [2+ 3+])
3	19877	0.997	0.915	0.592	3	([0+ 1+ 2+] 3+)
4	27180	0.828	0.828	0.436	3	([0− 1− 2−] 3+)
5	14602	0.978	0.985	0.654	4	(0− [1− 2− 3− 4−])
6	23340	0.974	0.809	0.684	3	(0− (1+ [2+ 3+]))
7	19853	0.967	0.825	0.703	3	(0− ([1+ 2+ 3+] 4+))
8	14790	0.994	0.994	0.808	3	([0− 1− 2− 3−] 4− 5−)
9	26244	0.971	0.996	0.817	3	(0− [1− 2− 3−])
10	26243	0.903	0.909	0.757	4	(([0− 1−] 2−) 3−)
11	26476	0.925	0.998	0.791	3	(0+ [1+ 2+ 3+])
12	15297	0.991	0.945	0.866	3	(0− ([1− 2−] [3− 4+]))
13	22866	0.845	0.600	0.722	5	([0+ 1+] [2+ 3+])
14	26238	0.785	0.807	0.663	7	([0− 1−] 2− 3−)
15	18995	0.770	0.621	0.681	9	((0+ [1+ 2+]) 3+)
16	28119	0.559	0.559	0.478	5	(0− 1− 2− 3−)
17	25371	0.983	0.942	0.907	4	(0− ([1− 2−] 3−))
18	26231	0.974	0.802	0.909	4	((0+ [1+ 2+]) 3+)
19	14796	0.760	0.686	0.700	9	(0− 1− [2− 3−] 4−)
20	20007	0.999	0.999	0.960	3	(0− 1− 2− [3− 4−])
21	22299	0.929	0.929	0.891	3	(0− 1− 2− 3−)
22	19255	0.948	0.940	0.918	5	([0− 1−] [2− 3−] 4−)
23	20764	0.880	0.987	0.852	3	([0+ 1+ 2+] 3+)
24	21553	0.982	0.982	0.961	3	(0− [1− 2−] 3− 4−)
25	27851	1.000	1.000	0.982	3	(0+ [1+ 2+] 3+)
26	27427	0.866	0.866	0.866	3	(0+ [1+ 2+ 3+])
27	15467	1.000	1.000	1.000	3	([0− 1− 2− 3−] 4− 5−)
28	19610	0.998	0.998	0.998	3	(0− ([1− 2−] 3− 4−))
29	20078	0.982	0.945	0.982	3	(0+ (1+ [2+ 3+]))
30	22181	0.912	0.940	0.912	3	(([0+ 1+] 2+) 3+)
31	25612	0.997	0.888	0.997	4	([0− 1−] [2− 3−])
32	27177	0.945	0.945	0.945	3	(0− [1+ 2+ 3+])
33	8962	0.933	0.933	0.933	3	(0− 1− [2− 3− 4− 5− 6−])
34	27530	0.999	0.974	0.999	3	[[0− 1−] 2− 3−]
35	19256	0.779	0.737	0.798	8	(0− 1− 2− 3−)
36	21317	0.974	0.974	0.995	3	(0+ 1+ [2+ 3+ 4+])
37	19852	0.890	0.821	0.919	6	(0− [1− 2−] 3−)
38	27250	0.967	0.967	0.997	3	([0+ 1+] [2+ 3+])
39	30976	0.963	1.000	0.996	3	[0− 1+ [2+ 3+]]
40	28245	0.715	0.715	0.775	3	(0− 1− 2− 3−)
41	26257	0.933	0.988	0.999	3	((0+ [1+ 2+]) 3+)
42	14839	0.860	0.912	0.928	8	([0− 1−] 2− 3− 4−)
43	27207	0.748	0.749	0.824	7	(0− [1− 2−] 3+)
44	12685	0.917	0.917	0.994	3	([0− 1− 2−] 3− 4− 5−)
45	21268	0.876	0.876	0.956	4	([0+ 1+] [2+ 3+])
46	23083	0.832	0.916	0.912	5	(([0− 1−] 2−) 3−)
47	25597	0.810	0.817	0.892	5	(0+ [1+ 2+] 3+)
48	19005	0.902	0.997	0.997	3	((0+ 1+ 2+) 3+)
49	15035	0.623	0.671	0.732	4	((0+ ([1+ 2+] 3+)) 4+)
50	28547	0.828	0.828	0.961	7	(0− 1− 2− 3−)
51	25375	0.866	0.866	1.000	3	([0− 1−] 2− 3−)
52	20332	0.860	0.860	0.997	5	(0+ 1+ [2+ 3+])
53	26364	0.839	0.869	0.988	5	(0+ 1+ 2+ 3+)
54	19909	0.818	0.818	0.968	6	(0− [1− 2−] 3−)
55	20601	0.586	0.586	0.756	5	(0− 1− 2− 3−)
56	15391	0.784	0.784	0.999	3	(0− (1− [2− 3− [4− 5−]]))
57	14573	0.501	0.501	0.722	4	(0− 1− (2− [3− 4−]) 5−)
58	25554	0.721	0.971	0.971	3	(([0+ 1+] 2+) 3+)
59	19526	0.543	0.471	0.814	4	((0− [1− 2−]) 3−)

For each gene cluster (row in the table) we report the Pearson correlation versus the corresponding instance-abundance measure for each of the three genome rearrangement measures compared in our experiment: our proposed measure ($\textsf{Diverge}$), signed break-point distance ($\mathsf {d_{SBP}}$) as in Definition 8 and CREx reversals distance ($d_{reversals}$) [28]. The column denoted “Size” gives the number of genes in the gene cluster, and the column denoted “PQ-tree” gives the corresponding PQ-tree representing the gene cluster. The functional category annotations for each cluster are given in Tables 3 and 4

**Table 3:** The functional categories for the gene clusters described in Tables 2, based on their COG annotations

#	Gene Cluster ID	Functional category
1	19876	Carbohydrate transport and metabolism—transcription
2	21344	Signal transduction mechanisms—cell wall/membrane/envelope biogenesis—defense mechanisms
3	19877	Carbohydrate transport and metabolism—general function prediction only
4	27180	Carbohydrate transport and metabolism—transcription
5	14602	Inorganic ion transport and metabolism
6	23340	Transcription—inorganic ion transport and metabolism
7	19853	Transcription—carbohydrate transport and metabolism
8	14790	Amino acid transport and metabolism—lipid transport and metabolism
9	26244	Carbohydrate transport and metabolism
10	26243	Carbohydrate transport and metabolism—transcription
11	26476	Inorganic ion transport and metabolism
12	15297	Energy production and conversion—coenzyme transport and metabolism
13	22866	Signal transduction mechanisms—cell wall/membrane/envelope biogenesis
14	26238	Carbohydrate transport and metabolism—transcription
15	18995	Carbohydrate transport and metabolism—transcription
16	28119	Carbohydrate transport and metabolism
17	25371	Coenzyme transport and metabolism—amino acid transport and metabolism
18	26231	Carbohydrate transport and metabolism
19	14796	Amino acid transport and metabolism
20	20007	Amino acid transport and metabolism
21	22299	Lipid transport and metabolism
22	19255	Posttranslational modification, protein turnover, chaperones—amino acid transport and metabolism
23	20764	Carbohydrate transport and metabolism
24	21553	Carbohydrate transport and metabolism
25	27851	Cell wall/membrane/envelope biogenesis—inorganic ion transport and metabolism
26	27427	Transcription—inorganic ion transport and metabolism
27	15467	Energy production and conversion
28	19610	Lipid transport and metabolism—general function prediction only
29	20078	Cell wall/membrane/envelope biogenesis—general function prediction only
30	22181	Energy production and conversion—posttranslational modification, protein turnover, chaperones
31	25612	Amino acid transport and metabolism
32	27177	Carbohydrate transport and metabolism
33	8962	Signal transduction mechanisms—inorganic ion transport and metabolism
34	27530	Energy production and conversion—transcription
35	19256	Amino acid transport and metabolism
36	21317	General function prediction only—posttranslational modification, protein turnover, chaperones
37	19852	Carbohydrate transport and metabolism
38	27250	Energy production and conversion
39	30976	Transcription—cell wall/membrane/envelope biogenesis —secondary metabolites biosynthesis, transport and catabolism
40	28245	Coenzyme transport and metabolism—carbohydrate transport and metabolism
41	26257	Carbohydrate transport and metabolism
42	14839	Amino acid transport and metabolism
43	27207	Cell wall/membrane/envelope biogenesis—defense mechanisms—transcription
44	12685	Posttranslational modification, protein turnover, chaperones—general function prediction only
45	21268	Defense mechanisms—signal transduction mechanisms
46	23083	Carbohydrate transport and metabolism—general function prediction only
47	25597	Amino acid transport and metabolism—posttranslational modification, protein turnover, chaperones

The first 47 gene clusters (out of 59), this table is continued in Table 4

**Table 4:** The functional categories for the gene clusters described in Table 2, based on their COG annotations

#	Gene cluster ID	Functional Category
48	19005	Carbohydrate transport and metabolism
49	15035	Cell motility
50	28547	Cell wall/membrane/envelope biogenesis
51	25375	Coenzyme transport and metabolism
52	20332	Amino acid transport and metabolism—energy production and conversion
53	26364	Inorganic ion transport and metabolism
54	19909	Amino acid transport and metabolism
55	20601	Inorganic ion transport and metabolism—coenzyme transport and metabolism
56	15391	Amino acid transport and metabolism
57	14573	Transcription—carbohydrate transport and metabolism
58	25554	Amino acid transport and metabolism
59	19526	Cell motility

The last 12 gene clusters (out of 59), this table continues Table 3

## Footnotes

1 That is, the character in position *i* in *G* is the same as the character in position *j* in *H*.

2 *S*(*x*) and $S(x')$ are strings of colors (which are unique), thus there is only one gene mapping of *S*(*x*) and $S(x')$.

3 Weight of a set is the sum of weights of its vertices.

4 A *vertex cover* is a set $S \subseteq V$ such that every edge of *G* has at least one endpoint in *S*.

5 Note that uniqueness follows from our use of colors.

6 That is, given a P-node *x*, a subset $C \subseteq \textsf{children}(x)$, a string $S'$, bounds on the number of deletions from the tree *T* and the string $S'$, $d_T$ and $d_S$, respectively, and a set of derivations $\mathcal {D}{}$, it returns the number of all partial derivations of $x^{(C)}$ to every prefix of $S'$ for each combination $(k_T,k_s,pos,score)$ having exactly $k_T$ deletions from the tree, $k_S$ deletions from the string, *pos* leaves with positive sign, whose divergence value is at most *score*, and that use $\gamma$ children from *C* (including repetitions).
